# Supplementary material for: Generating global network structures by triad types
Source: PLoS One. 2018 May 30;13(5):e0197514. doi: 10.1371/journal.pone.0197514 (PMC5976167; doi:10.1371/journal.pone.0197514)
Supplement: S1 Appendix — (PDF) [file pone.0197514.s001.pdf]

S1 Appendix: Generating totally randomised networks and networks with a given level of errors

Totally randomised networks can be generated based on an ideal network: k links in complete blocks are randomly chosen and replaced by non-links. At the same time, k non-links are randomly chosen and replaced with links. In other words, the number of links is relocated in such a way that the overall density in complete blocks and overall density in null blocks are equal (i.e., all expected densities of all the blocks in the blockmodel are equal). The number of relocated links k is calculated as

$$k = m - \frac{m^2}{n^2 - n} \tag{1}$$

where m is the number of links and n is the number of units in a selected type of blockmodel.

Instead of totally randomised networks, blockmodels with a certain level of errors can be analysed. In such case, when a network with a given blockmodel structure has to be generated with a certain level of errors, the number of relocated links is calculated as

$$k = m - \left(\frac{m^2}{n^2 - n}\right) * LE \tag{2}$$

where the level of errors (LE) can take a value on the interval $[0, 1]$ (0 stands for an ideal network and 1 for a random network).
