# Supplementary material for: Generating global network structures by triad types
Source: PLoS One. 2018 May 30;13(5):e0197514. doi: 10.1371/journal.pone.0197514 (PMC5976167; doi:10.1371/journal.pone.0197514)

S2 Appendix: Selected allowed and forbidden triad types

The sets of allowed and forbidden triad types can be further reduced to selected allowed and selected forbidden triad types. This means that not all possible or all allowed or all forbidden types of triads are considered. One could choose the most appropriate terms based on observations of the A-measure. To obtain a better triad selection, it is beneficial to observe the A-values for networks with different levels of errors.

Here, the most common (and uncommon) triads for each blockmodel type can be recognised by their sensitivity to different levels of errors. The idea is as follows: the most important triads are those with the highest absolute A-measure values for all levels of errors and with as close to a linear trend as possible through all levels of errors, indicating that a certain triad is not greatly affected by the level of errors (see Fig 1 for the visualised relationship between the level of errors and A-measure for different blockmodel types and triad types).

For some triads, the A-measure values are nearly constant for all levels of errors. Such a triad is triad type 300 in the case of a transitivity blockmodel with complete blocks on the diagonal. The value of the A-measure for these triad types is not associated with the level of errors. On the other hand, for many triad types a sharp change in the A-measure value at a certain level of errors is common. For example, in the case of a hierarchical blockmodel without complete blocks on the diagonal, the value of the A-measure for triads of types 012, 111D, 111U, 030T, 030C and 210 is zero in the case of an ideal network while it approaches 1 at very low levels of errors (i.e. between 0.2 and 0.4) and then remains constant. Some values first increase very fast at low levels of errors and then decrease at higher levels of errors. One example is the number of compete subgraphs of size three with one missing link in a cohesive blockmodel.

The values of the A-measure for some types of triads are increasing or decreasing nearly linearly with the level of errors. These types of triads can be seen as triads that should be considered when generating networks with a given blockmodel structure. However, these types of triads can be further differentiated. For example, there are many types of triads with similar A-measure values for different levels of errors within some types of blockmodels. This could indicate that certain types of triads are defined similarly and are therefore not needed when generating networks with a given blockmodel structure.

Some types of triads which are strongly influenced by the level of errors at low levels and less influenced by the level of errors at high level of errors (and vice versa) could also be chosen. In this case, it may happen that one should choose different types of triads for networks with higher and for networks with lower levels of errors.

Fig 1. A-measure values (y-axis) for different levels of errors (x-axis) and different types of blockmodels (by rows: A = cohesive; B = symmetric core-periphery; C = asymmetric core-periphery; D = hierarchical without complete blocks on the diagonal; E = hierarchical with complete blocks on the diagonal; F = transitivity without complete blocks on the diagonal; G = transitivity with complete blocks on the diagonal)

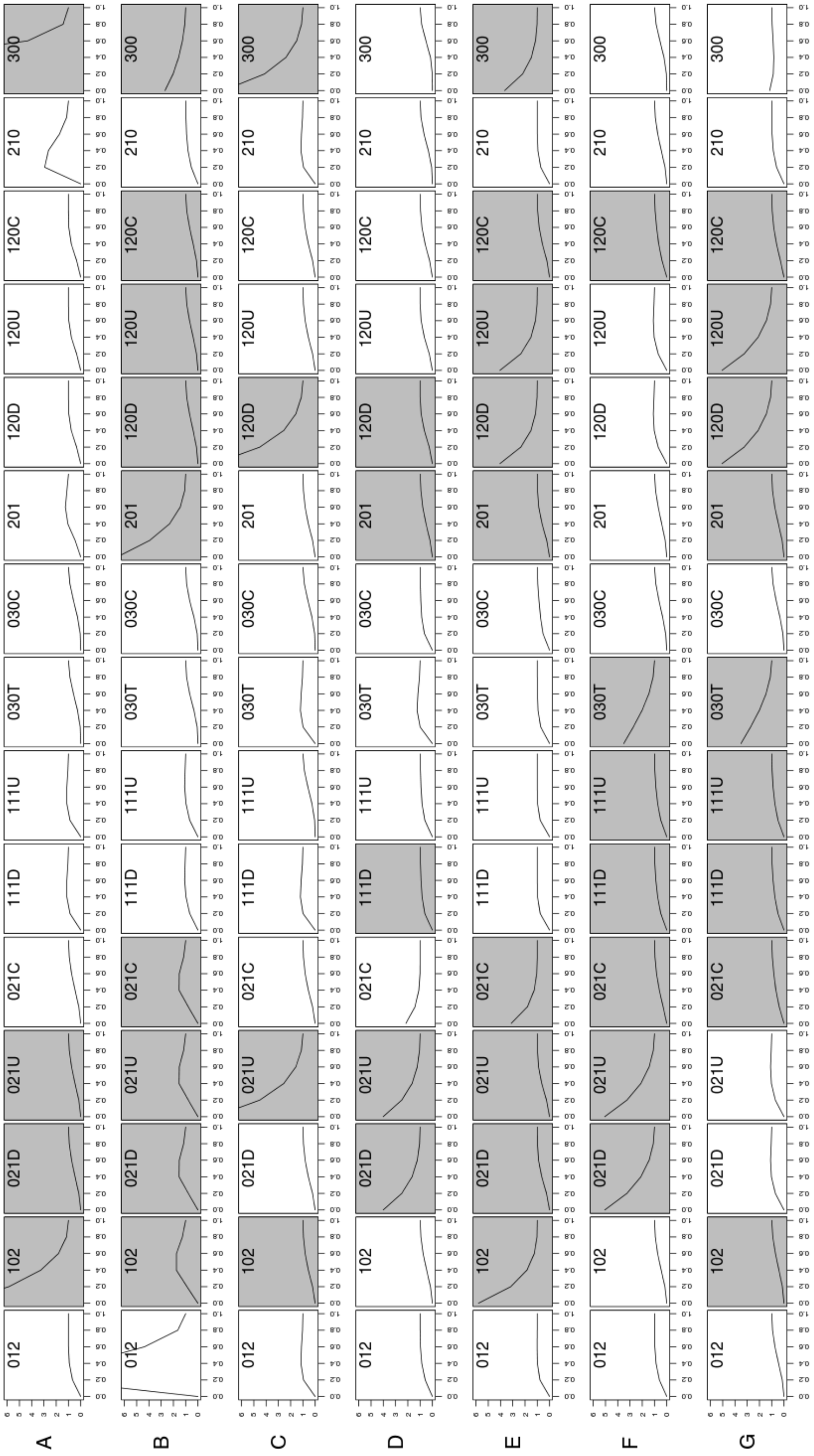

Supplement: S2 Appendix — (PDF) [file pone.0197514.s002.pdf]
