# Supplementary figures and images for: Generating global network structures by triad types
Source: PLoS One. 2018 May 30;13(5):e0197514. doi: 10.1371/journal.pone.0197514 (PMC5976167; doi:10.1371/journal.pone.0197514)

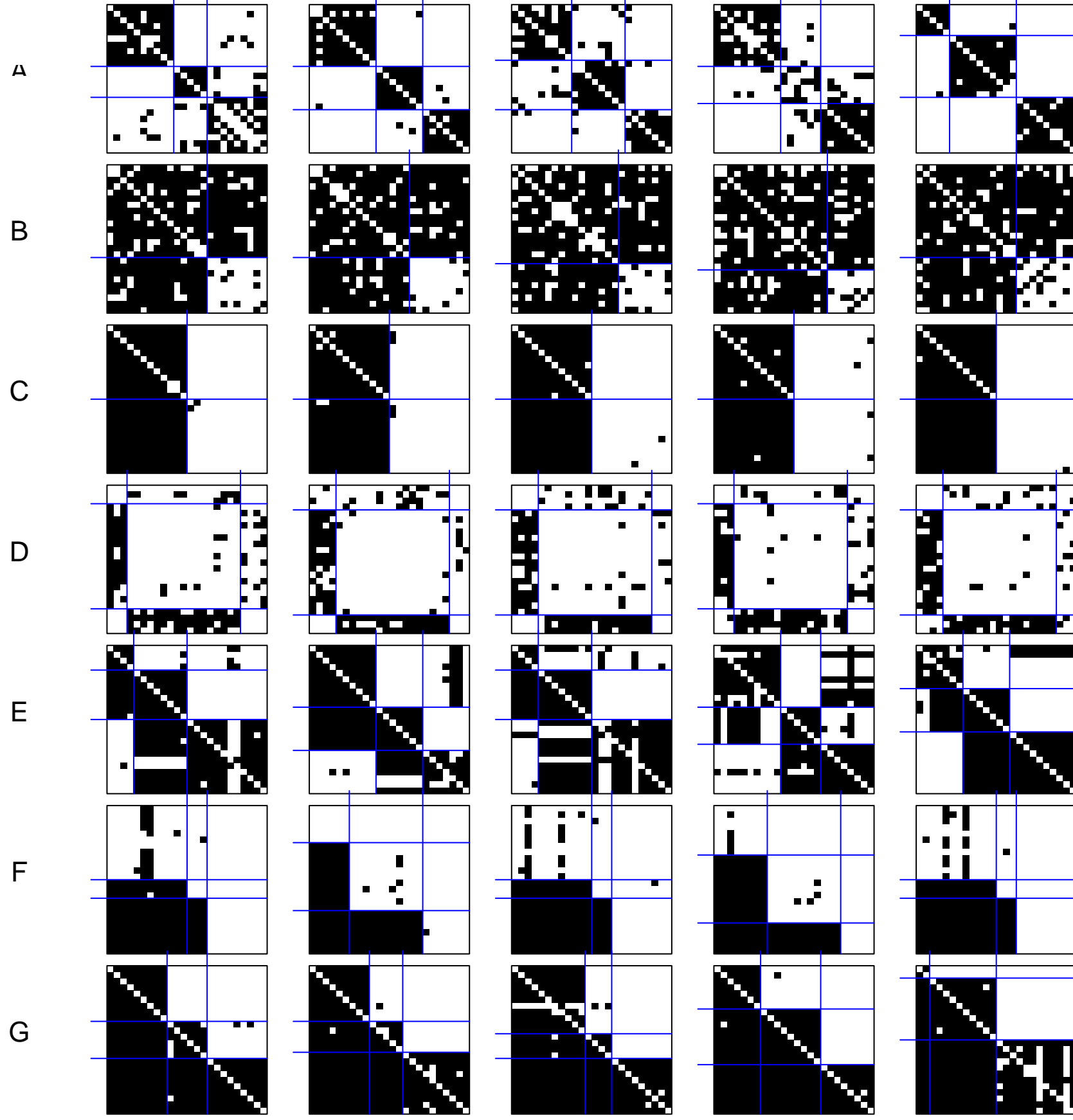

Supplement: S1 Fig — By rows: (A) cohesive; (B) symmetric core-periphery; (C) asymmetric core-periphery; (D) hierarchical without complete blocks on the diagonal; (E) hierarchical with complete blocks on the diagonal; (F) transitivity without complete blocks on the diagonal; (G) transitivity with complete blocks on the diagonal. (PDF) [file pone.0197514.s003.pdf]

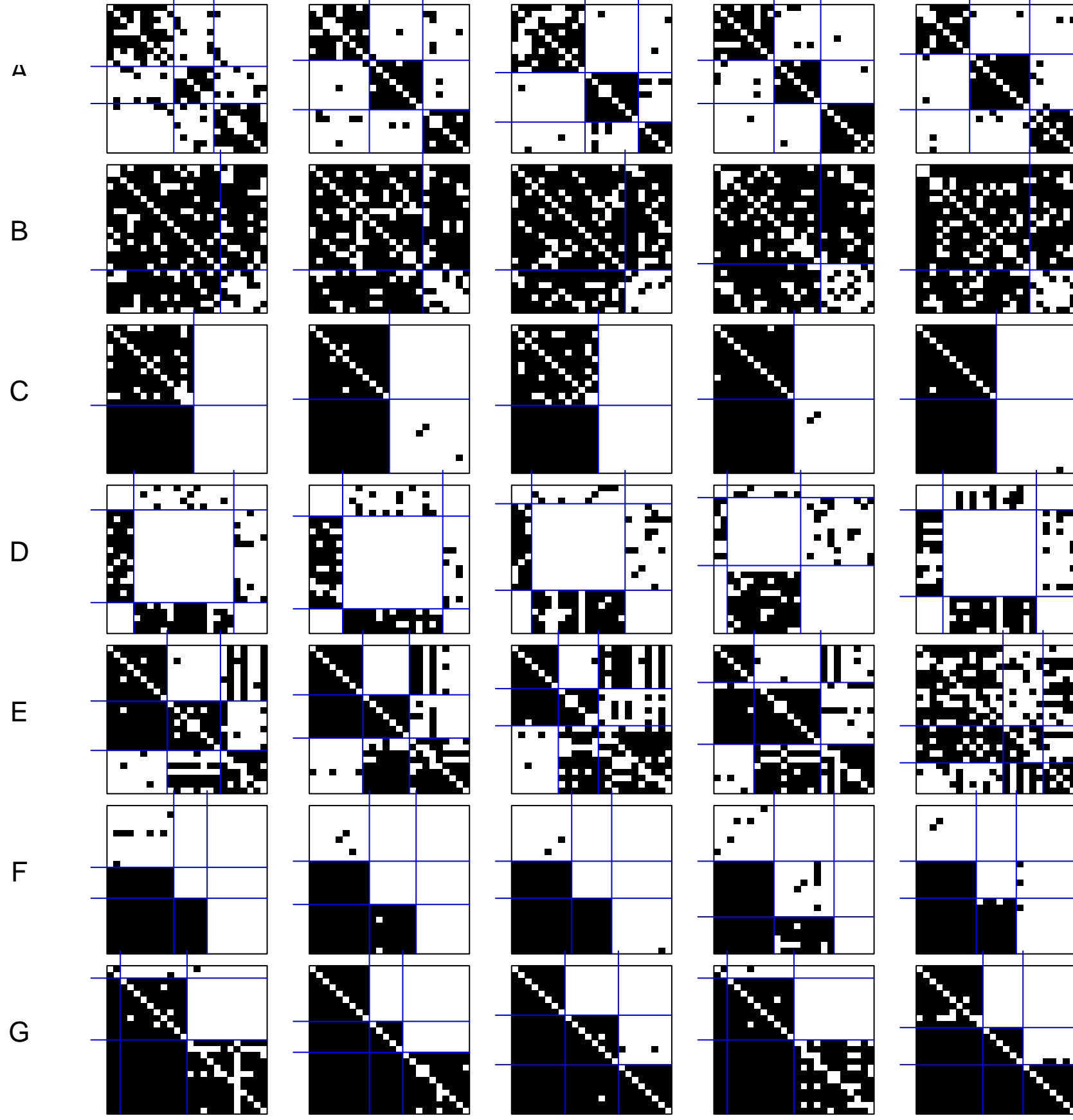

Supplement: S2 Fig — By rows: (A) cohesive; (B) symmetric core-periphery; (C) asymmetric core-periphery; (D) hierarchical without complete blocks on the diagonal; (E) hierarchical with complete blocks on the diagonal; (F) transitivity without complete blocks on the diagonal; (G) transitivity with complete blocks on the diagonal. (PDF) [file pone.0197514.s004.pdf]

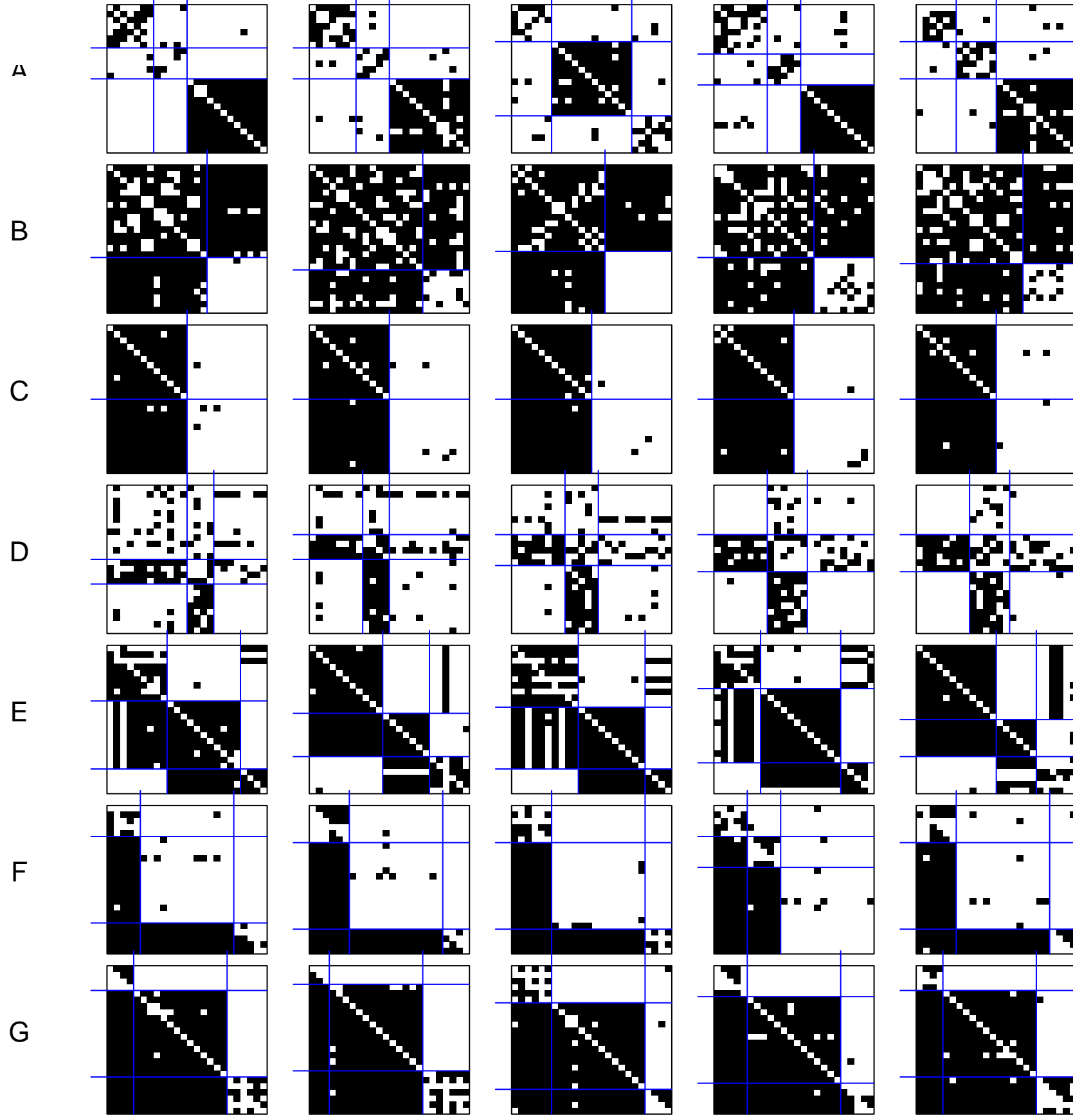

Supplement: S3 Fig — By rows: (A) cohesive; (B) symmetric core-periphery; (C) asymmetric core-periphery; (D) hierarchical without complete blocks on the diagonal; (E) hierarchical with complete blocks on the diagonal; (F) transitivity without complete blocks on the diagonal; (G) transitivity with complete blocks on the diagonal. (PDF) [file pone.0197514.s005.pdf]

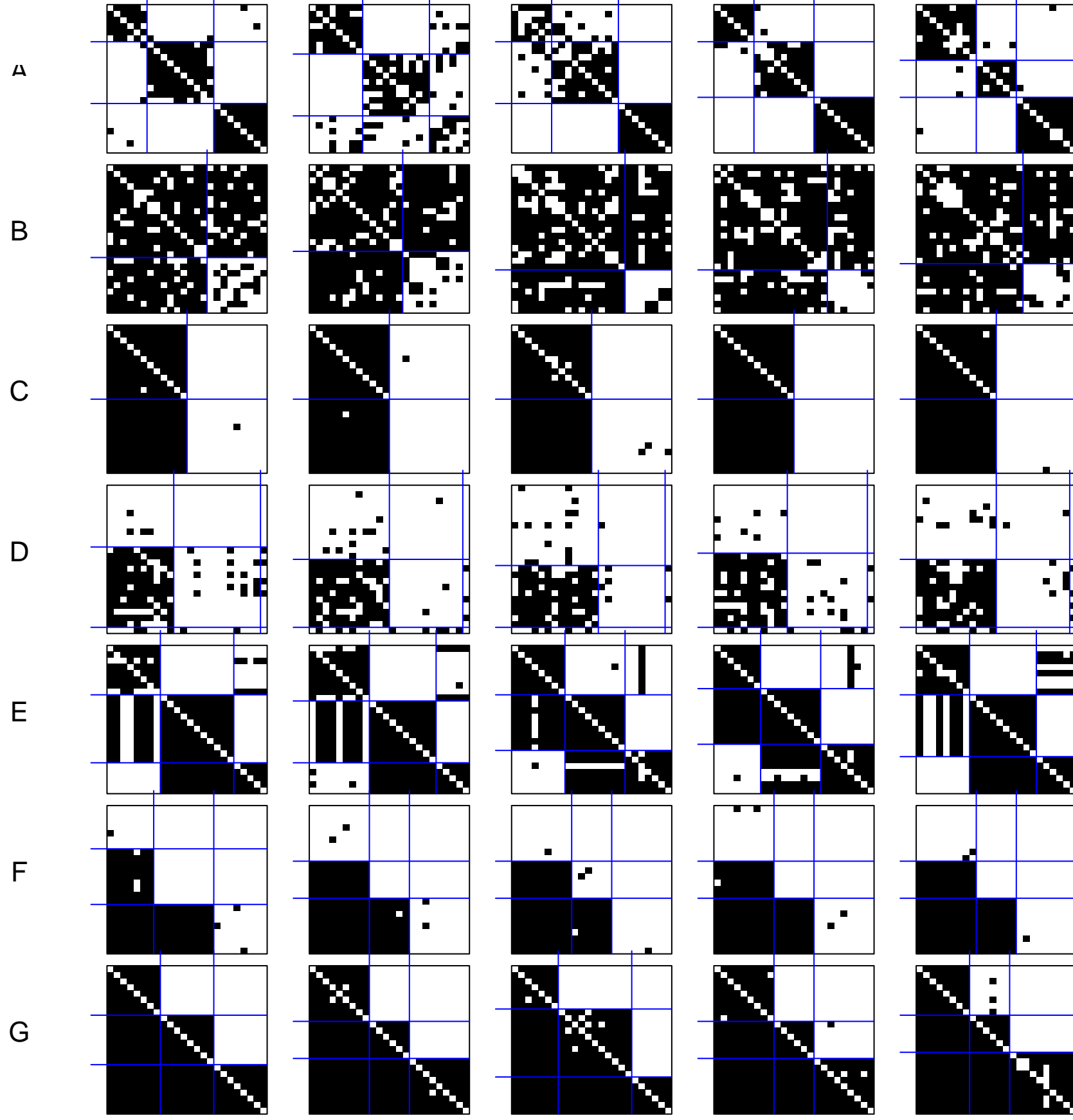

Supplement: S4 Fig — By rows: (A) cohesive; (B) symmetric core-periphery; (C) asymmetric core-periphery; (D) hierarchical without complete blocks on the diagonal; (E) hierarchical with complete blocks on the diagonal; (F) transitivity without complete blocks on the diagonal; (G) transitivity with complete blocks on the diagonal. (PDF) [file pone.0197514.s006.pdf]

A

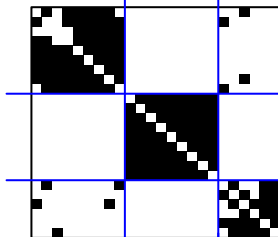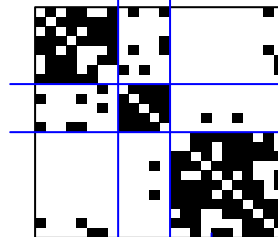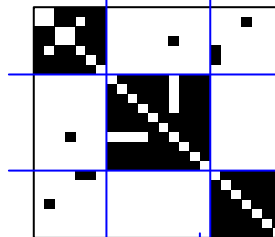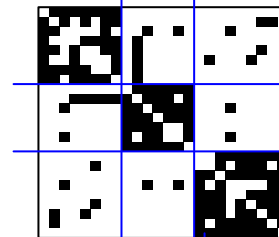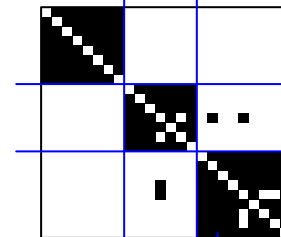

B

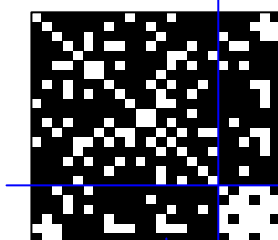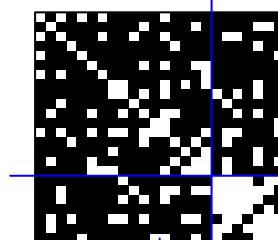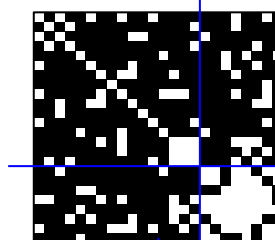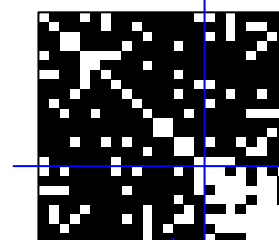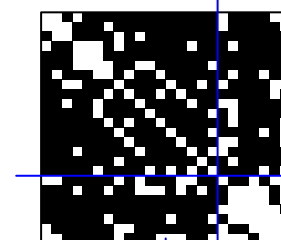

C

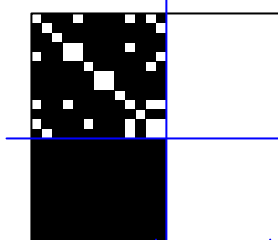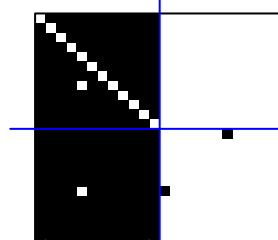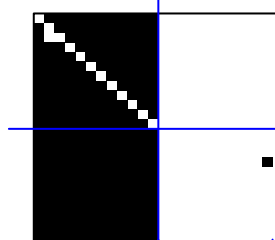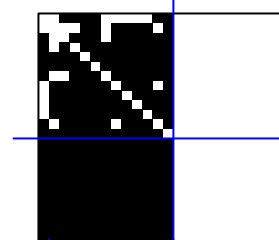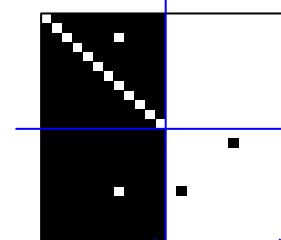

D

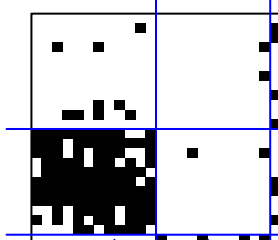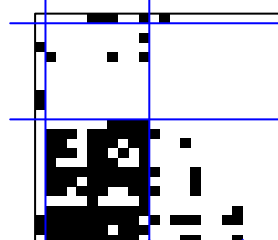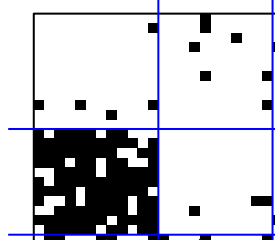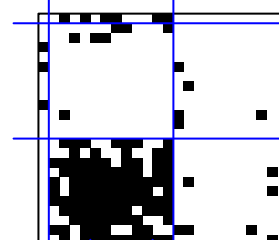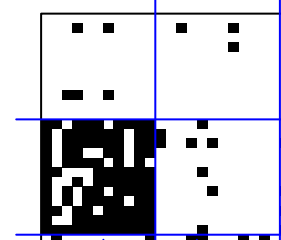

E

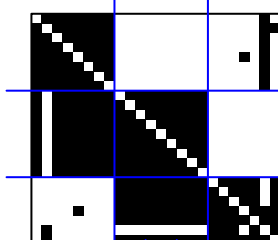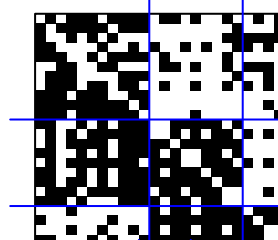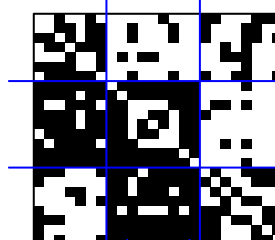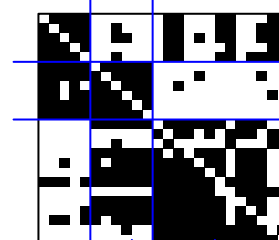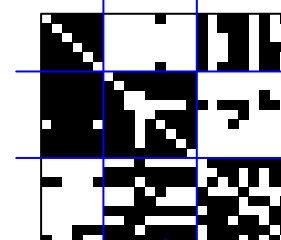

F

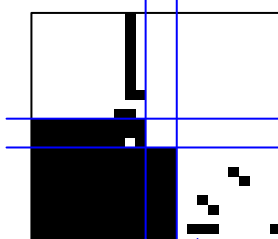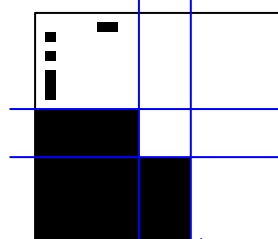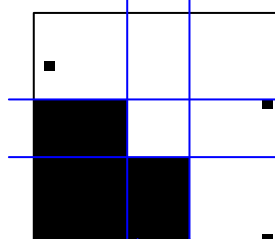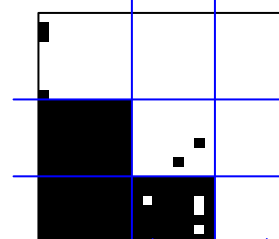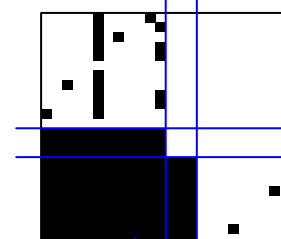

G

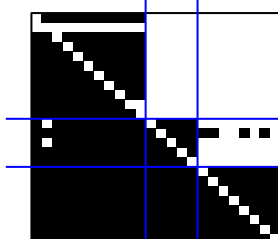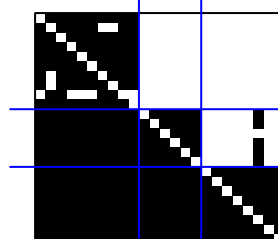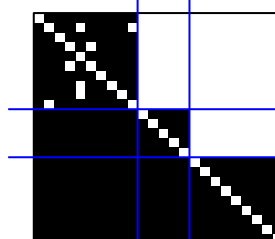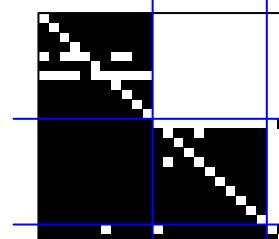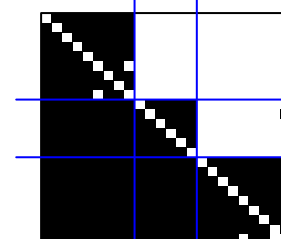

Supplement: S5 Fig — By rows: (A) cohesive; (B) symmetric core-periphery; (C) asymmetric core-periphery; (D) hierarchical without complete blocks on the diagonal; (E) hierarchical with complete blocks on the diagonal; (F) transitivity without complete blocks on the diagonal; (G) transitivity with complete blocks on the diagonal. (PDF) [file pone.0197514.s007.pdf]

A

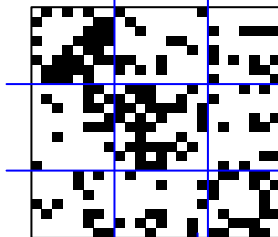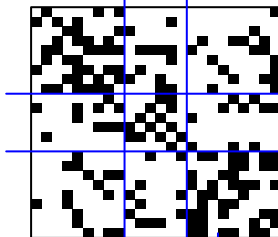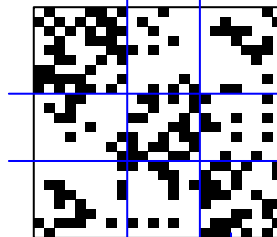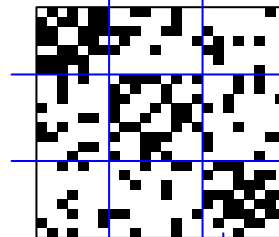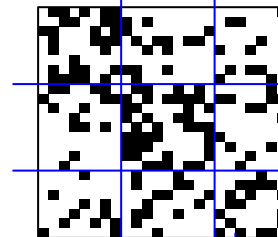

B

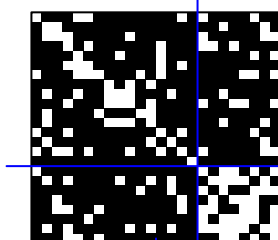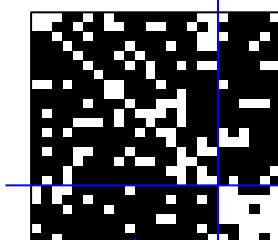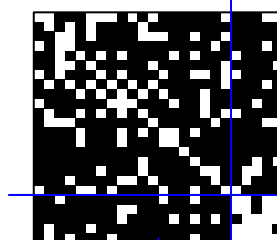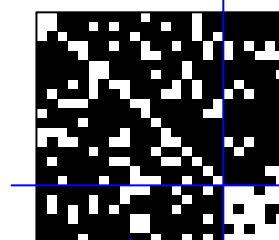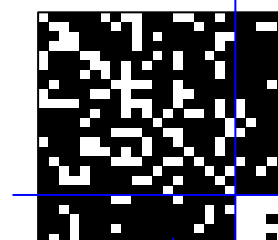

C

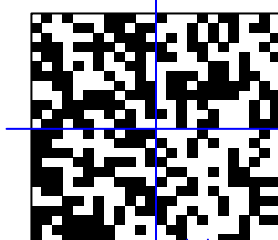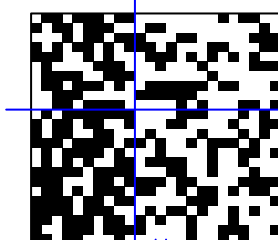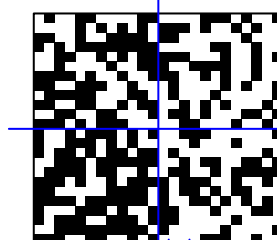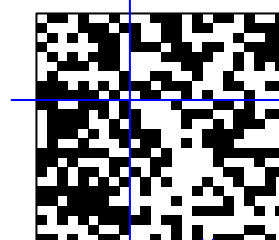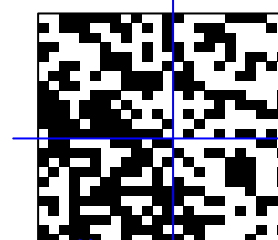

D

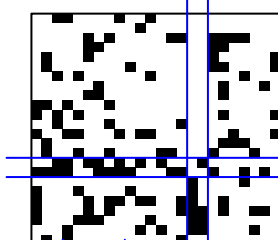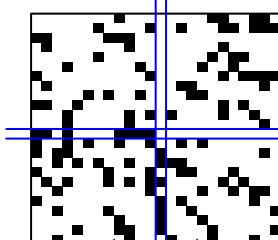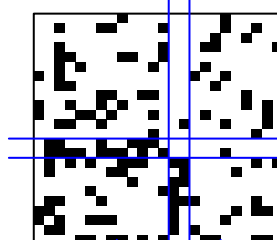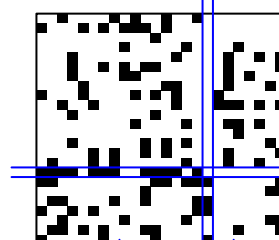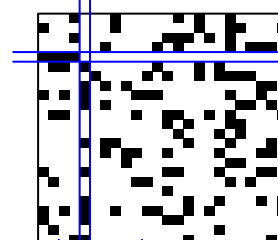

E

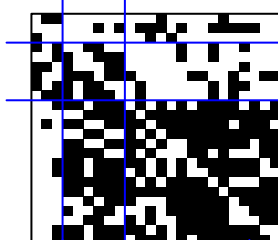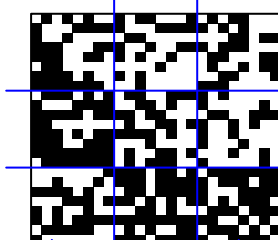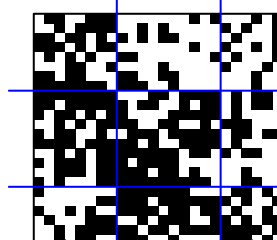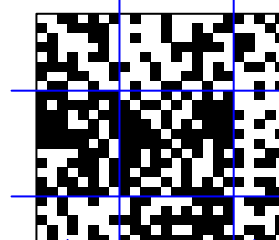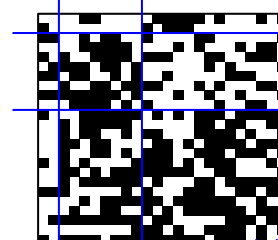

F

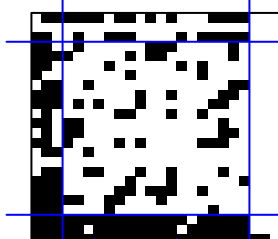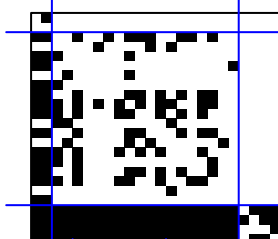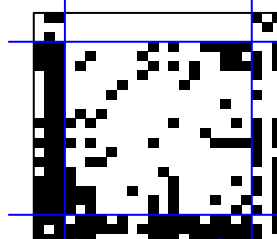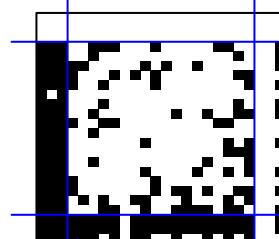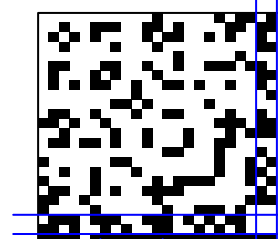

G

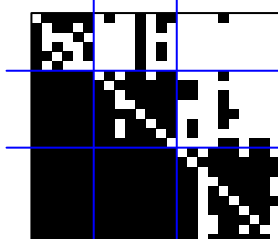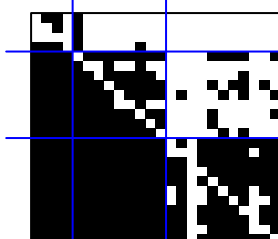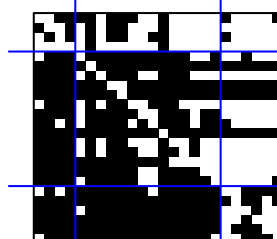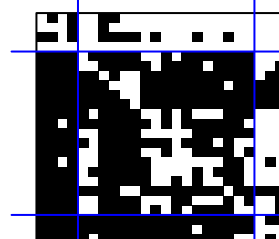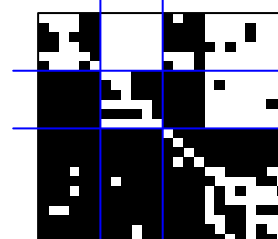

Supplement: S6 Fig — By rows: (A) cohesive; (B) symmetric core-periphery; (C) asymmetric core-periphery; (D) hierarchical without complete blocks on the diagonal; (E) hierarchical with complete blocks on the diagonal; (F) transitivity without complete blocks on the diagonal; (G) transitivity with complete blocks on the diagonal. (PDF) [file pone.0197514.s008.pdf]

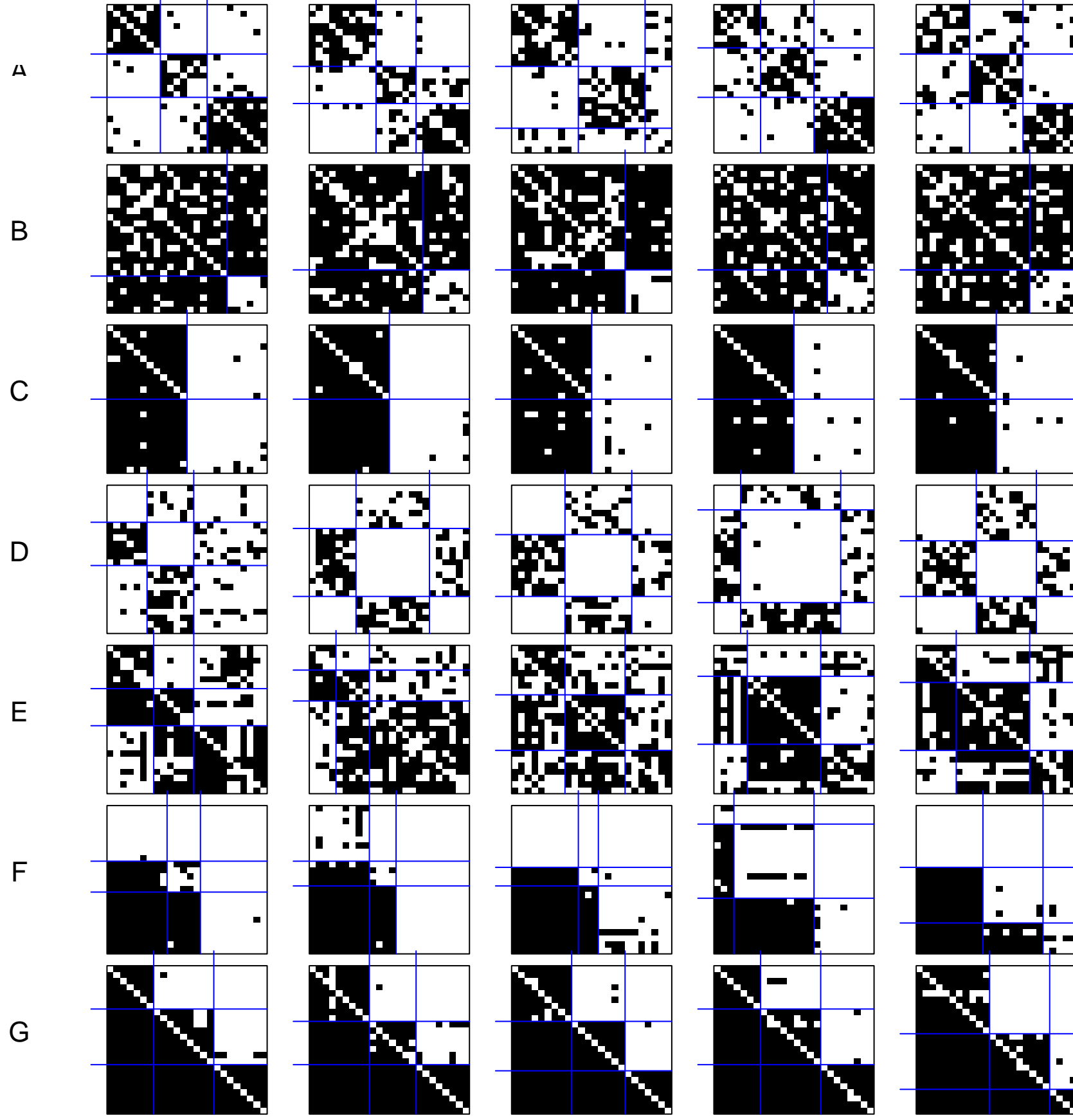

Supplement: S7 Fig — By rows: (A) cohesive; (B) symmetric core-periphery; (C) asymmetric core-periphery; (D) hierarchical without complete blocks on the diagonal; (E) hierarchical with complete blocks on the diagonal; (F) transitivity without complete blocks on the diagonal; (G) transitivity with complete blocks on the diagonal. (PDF) [file pone.0197514.s009.pdf]

A

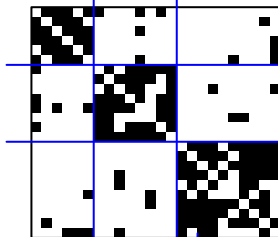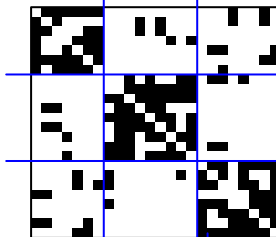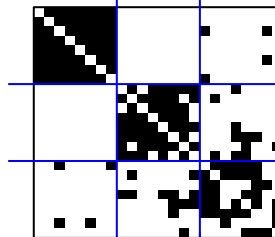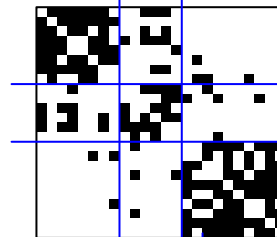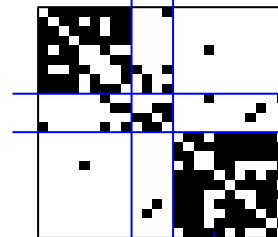

B

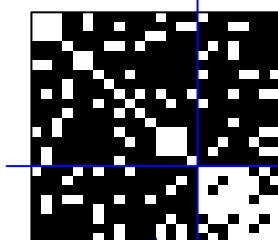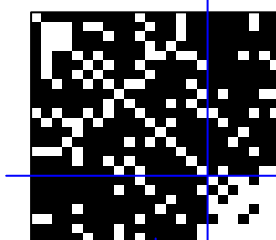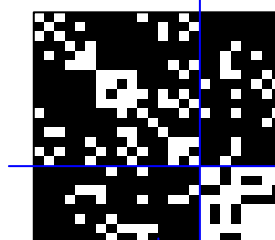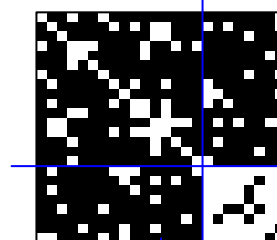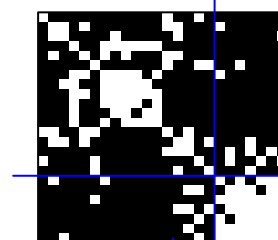

C

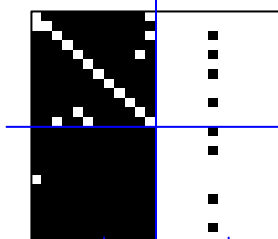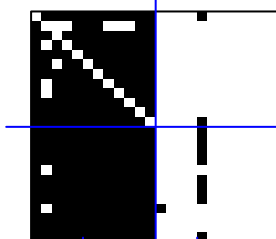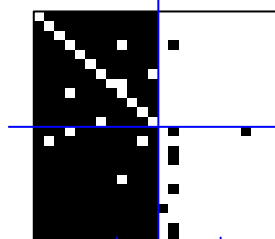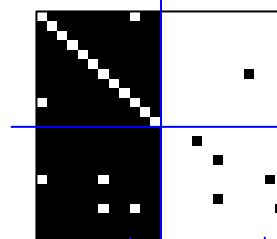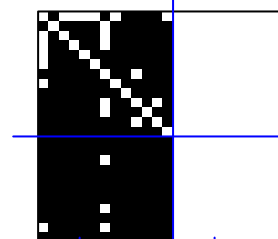

D

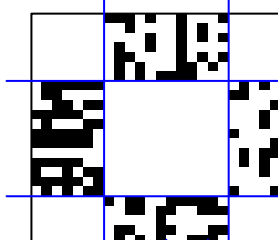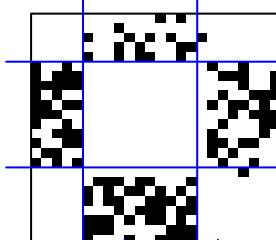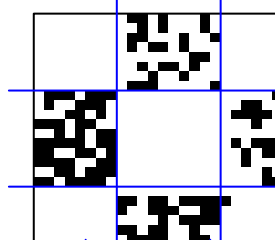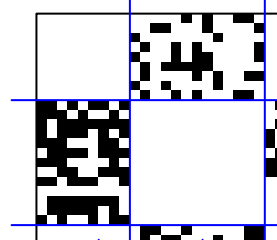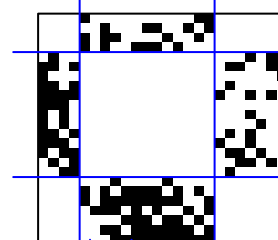

E

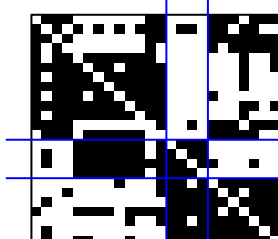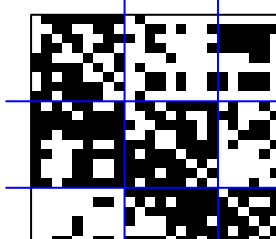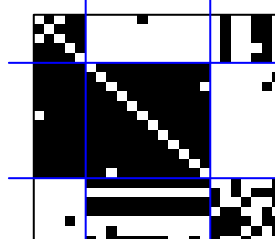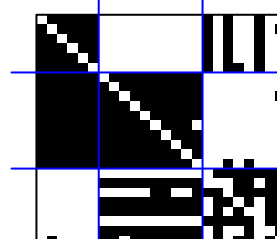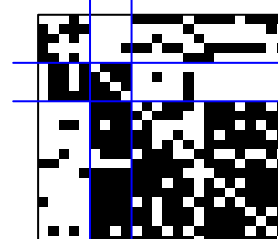

F

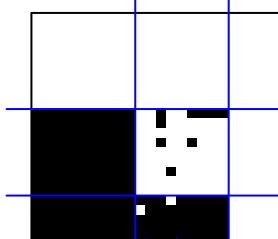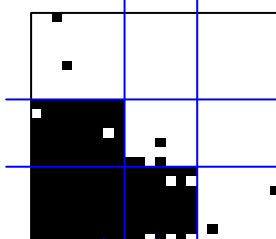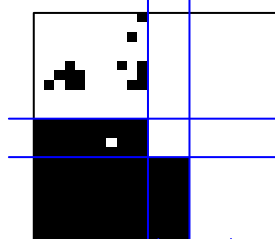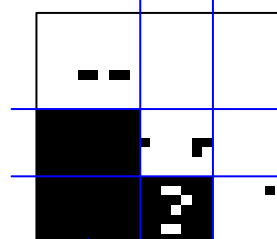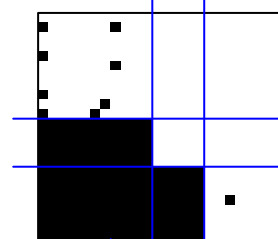

G

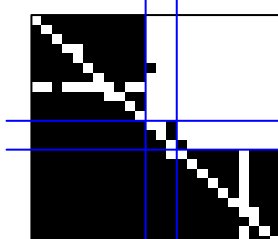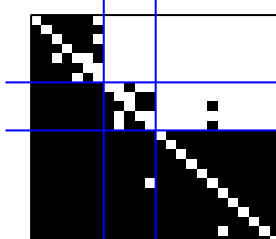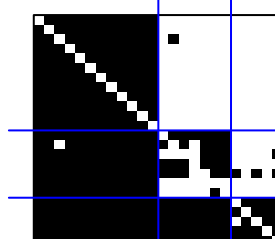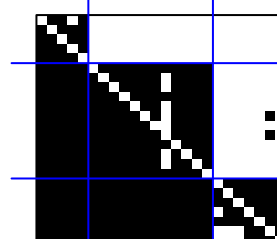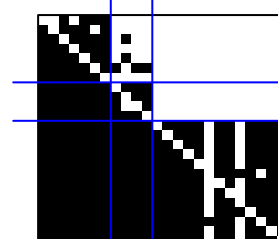

Supplement: S8 Fig — By rows: (A) cohesive; (B) symmetric core-periphery; (C) asymmetric core-periphery; (D) hierarchical without complete blocks on the diagonal; (E) hierarchical with complete blocks on the diagonal; (F) transitivity without complete blocks on the diagonal; (G) transitivity with complete blocks on the diagonal. (PDF) [file pone.0197514.s010.pdf]

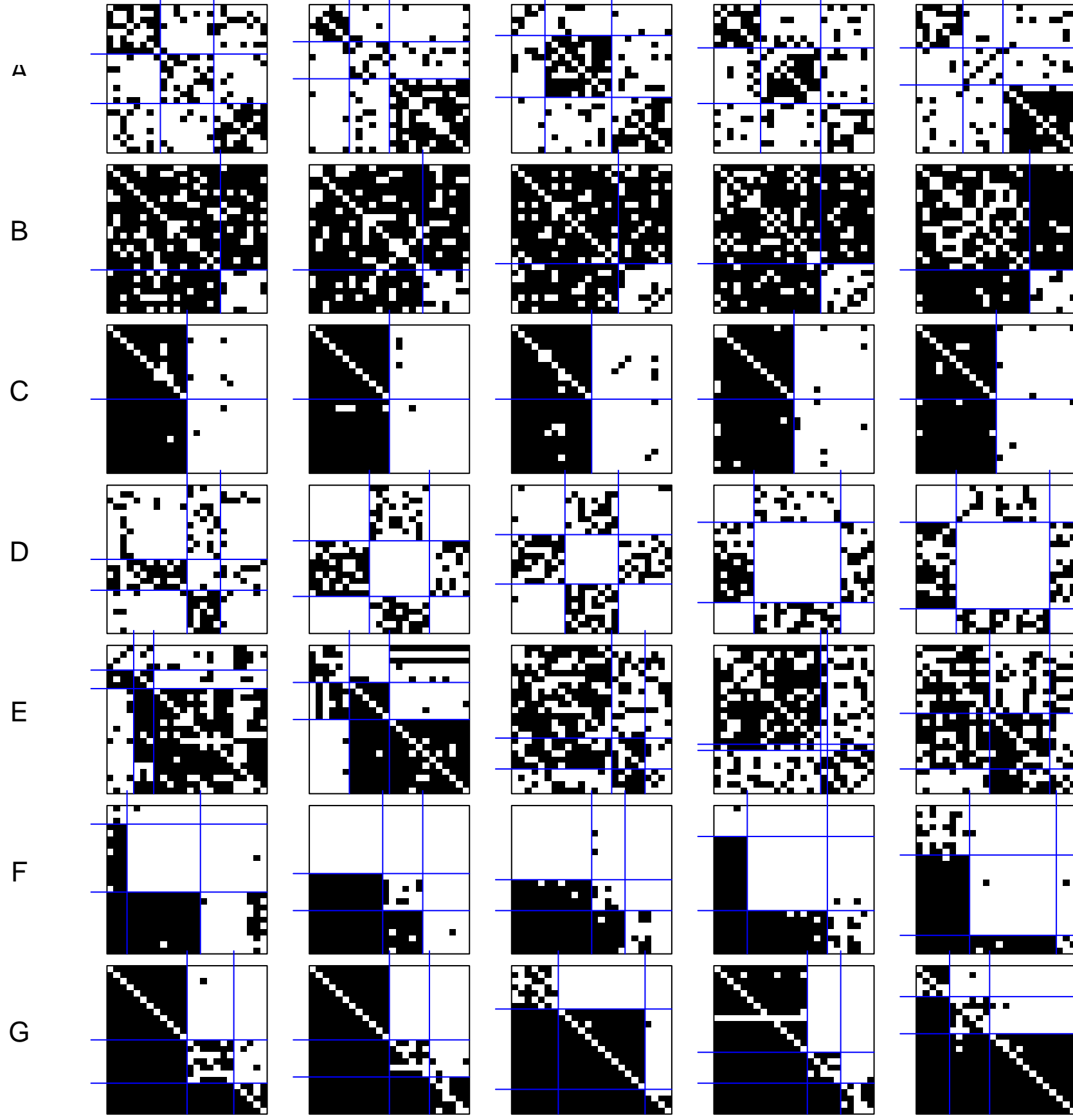

Supplement: S9 Fig — By rows: (A) cohesive; (B) symmetric core-periphery; (C) asymmetric core-periphery; (D) hierarchical without complete blocks on the diagonal; (E) hierarchical with complete blocks on the diagonal; (F) transitivity without complete blocks on the diagonal; (G) transitivity with complete blocks on the diagonal. (PDF) [file pone.0197514.s011.pdf]

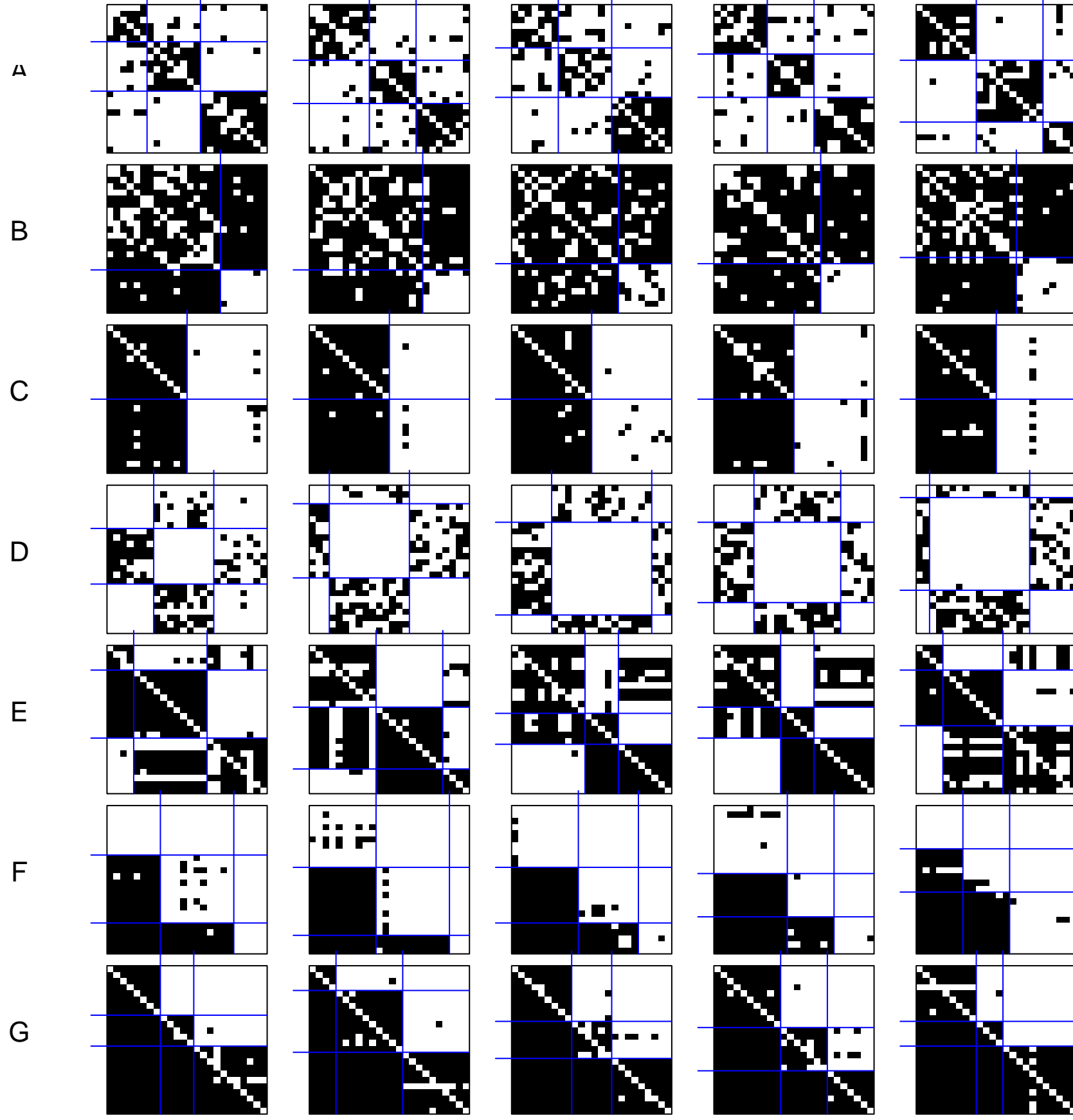

Supplement: S10 Fig — By rows: (A) cohesive; (B) symmetric core-periphery; (C) asymmetric core-periphery; (D) hierarchical without complete blocks on the diagonal; (E) hierarchical with complete blocks on the diagonal; (F) transitivity without complete blocks on the diagonal; (G) transitivity with complete blocks on the diagonal. (PDF) [file pone.0197514.s012.pdf]

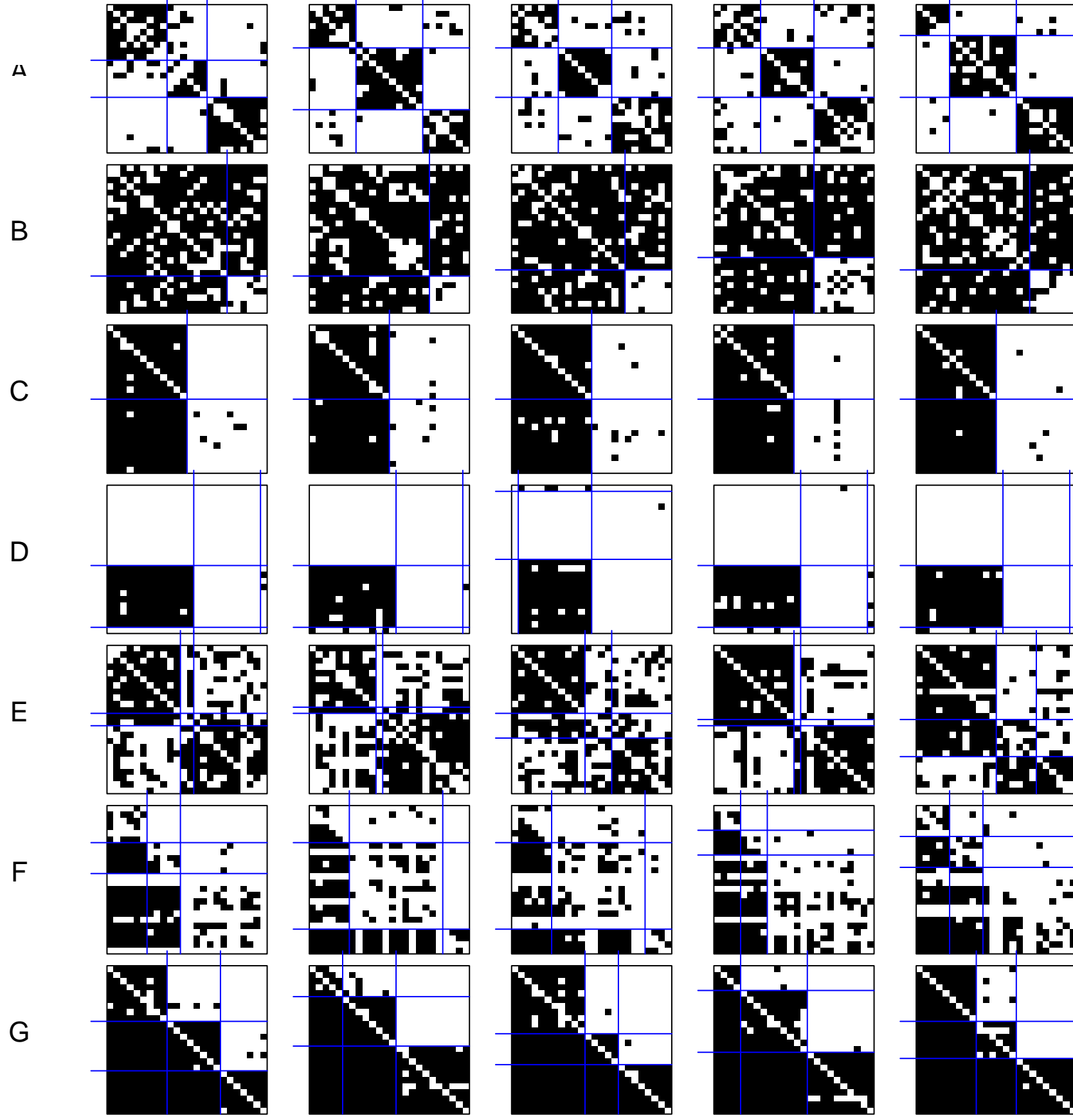

Supplement: S11 Fig — By rows: (A) cohesive; (B) symmetric core-periphery; (C) asymmetric core-periphery; (D) hierarchical without complete blocks on the diagonal; (E) hierarchical with complete blocks on the diagonal; (F) transitivity without complete blocks on the diagonal; (G) transitivity with complete blocks on the diagonal. (PDF) [file pone.0197514.s013.pdf]

A

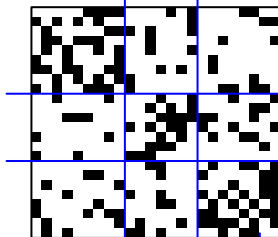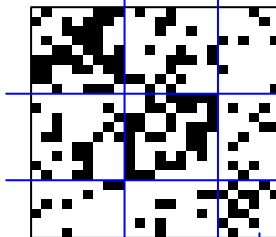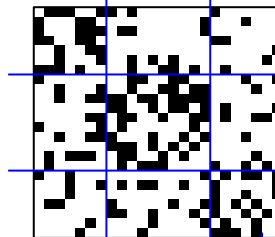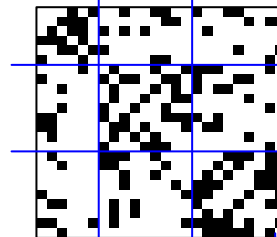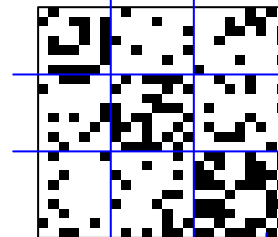

B

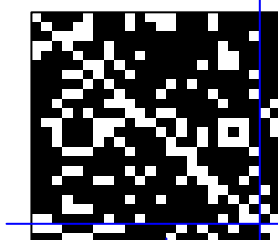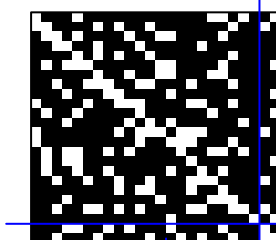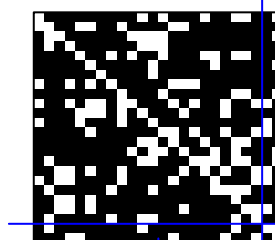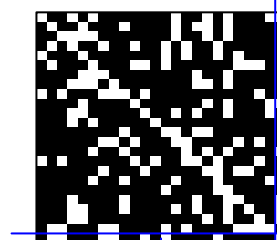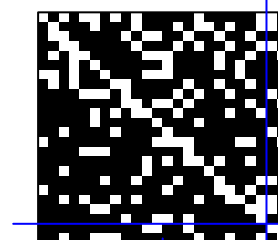

C

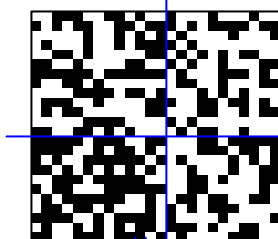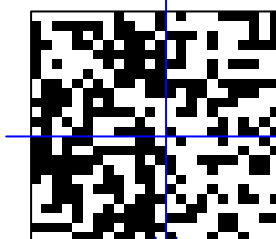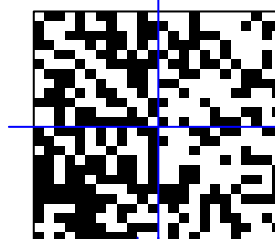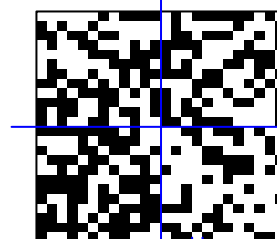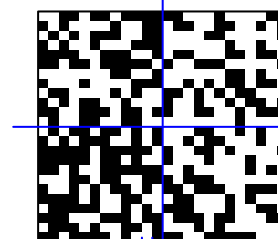

D

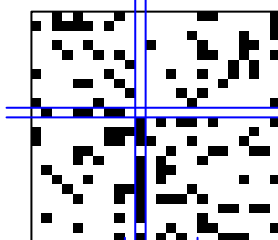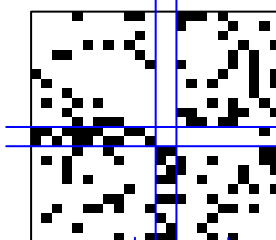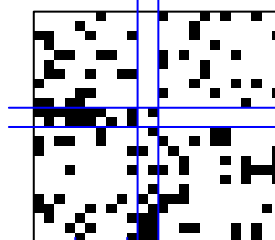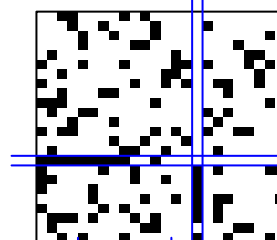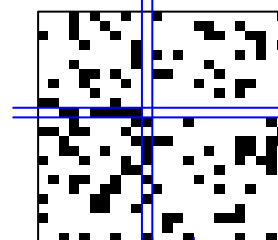

E

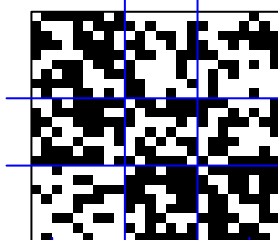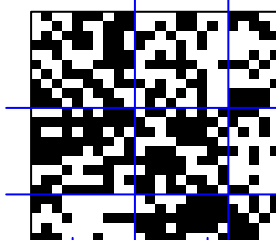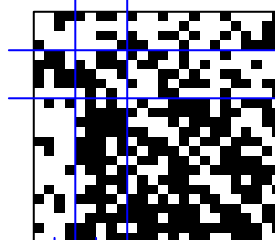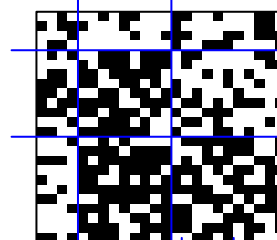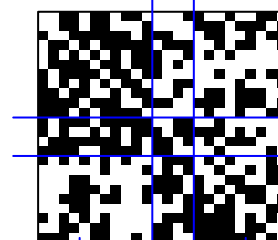

F

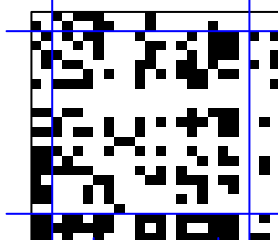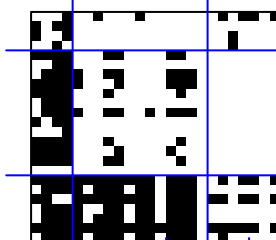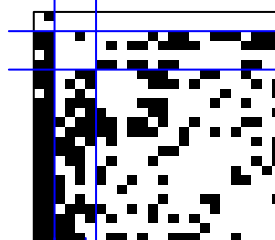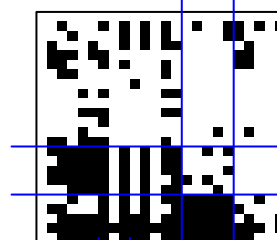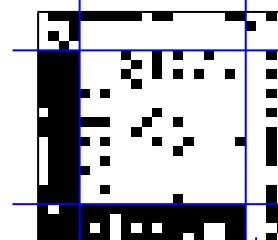

G

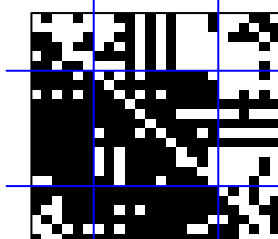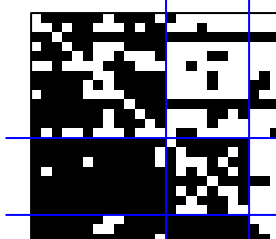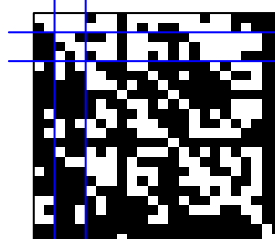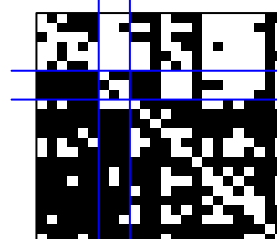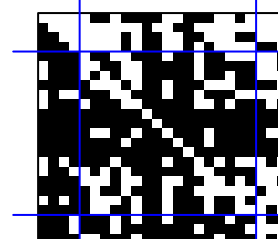

Supplement: S12 Fig — By rows: (A) cohesive; (B) symmetric core-periphery; (C) asymmetric core-periphery; (D) hierarchical without complete blocks on the diagonal; (E) hierarchical with complete blocks on the diagonal; (F) transitivity without complete blocks on the diagonal; (G) transitivity with complete blocks on the diagonal. (PDF) [file pone.0197514.s014.pdf]

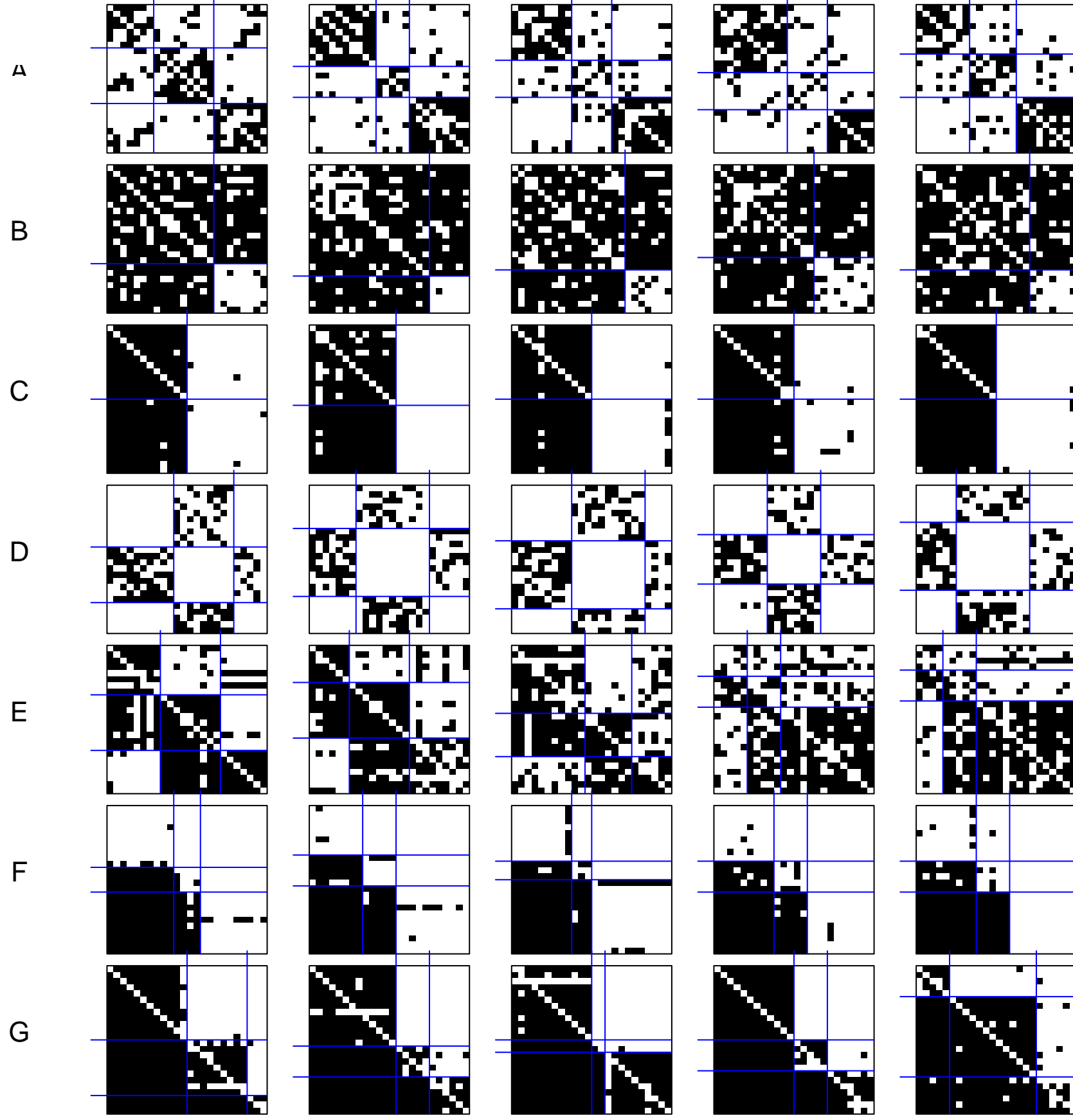

Supplement: S13 Fig — By rows: (A) cohesive; (B) symmetric core-periphery; (C) asymmetric core-periphery; (D) hierarchical without complete blocks on the diagonal; (E) hierarchical with complete blocks on the diagonal; (F) transitivity without complete blocks on the diagonal; (G) transitivity with complete blocks on the diagonal. (PDF) [file pone.0197514.s015.pdf]

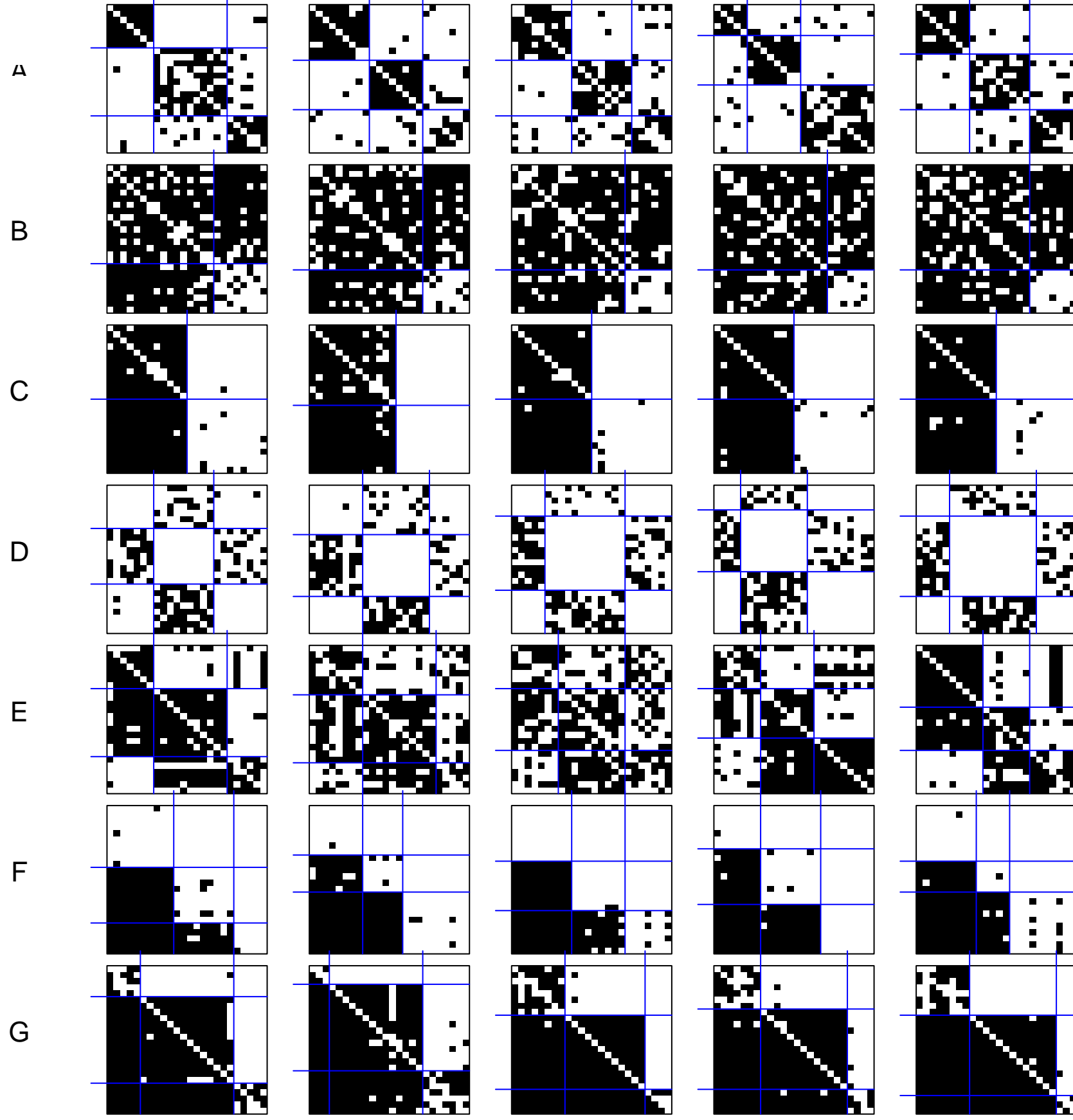

Supplement: S14 Fig — By rows: (A) cohesive; (B) symmetric core-periphery; (C) asymmetric core-periphery; (D) hierarchical without complete blocks on the diagonal; (E) hierarchical with complete blocks on the diagonal; (F) transitivity without complete blocks on the diagonal; (G) transitivity with complete blocks on the diagonal. (PDF) [file pone.0197514.s016.pdf]

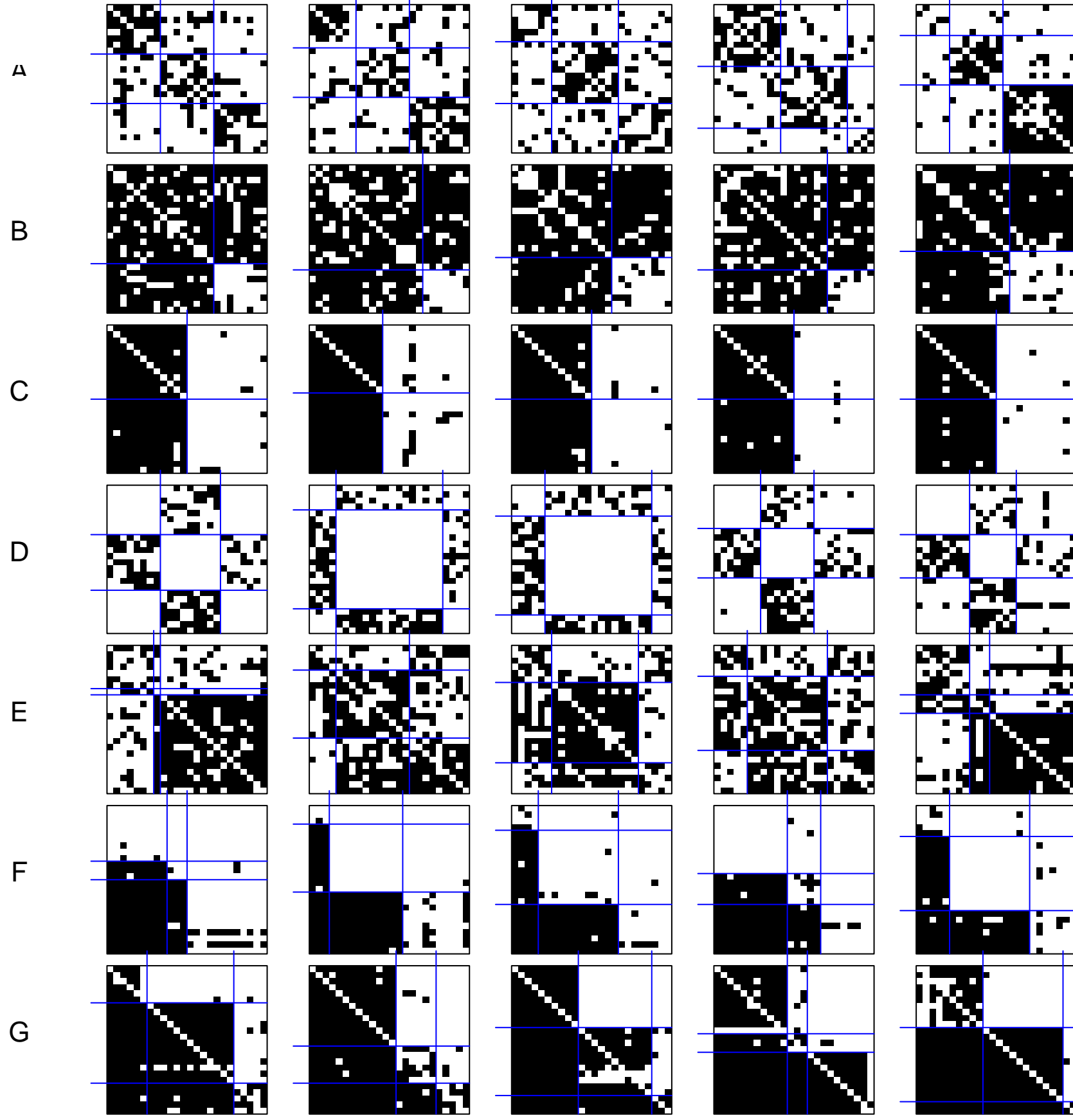

Supplement: S15 Fig — By rows: (A) cohesive; (B) symmetric core-periphery; (C) asymmetric core-periphery; (D) hierarchical without complete blocks on the diagonal; (E) hierarchical with complete blocks on the diagonal; (F) transitivity without complete blocks on the diagonal; (G) transitivity with complete blocks on the diagonal. (PDF) [file pone.0197514.s017.pdf]

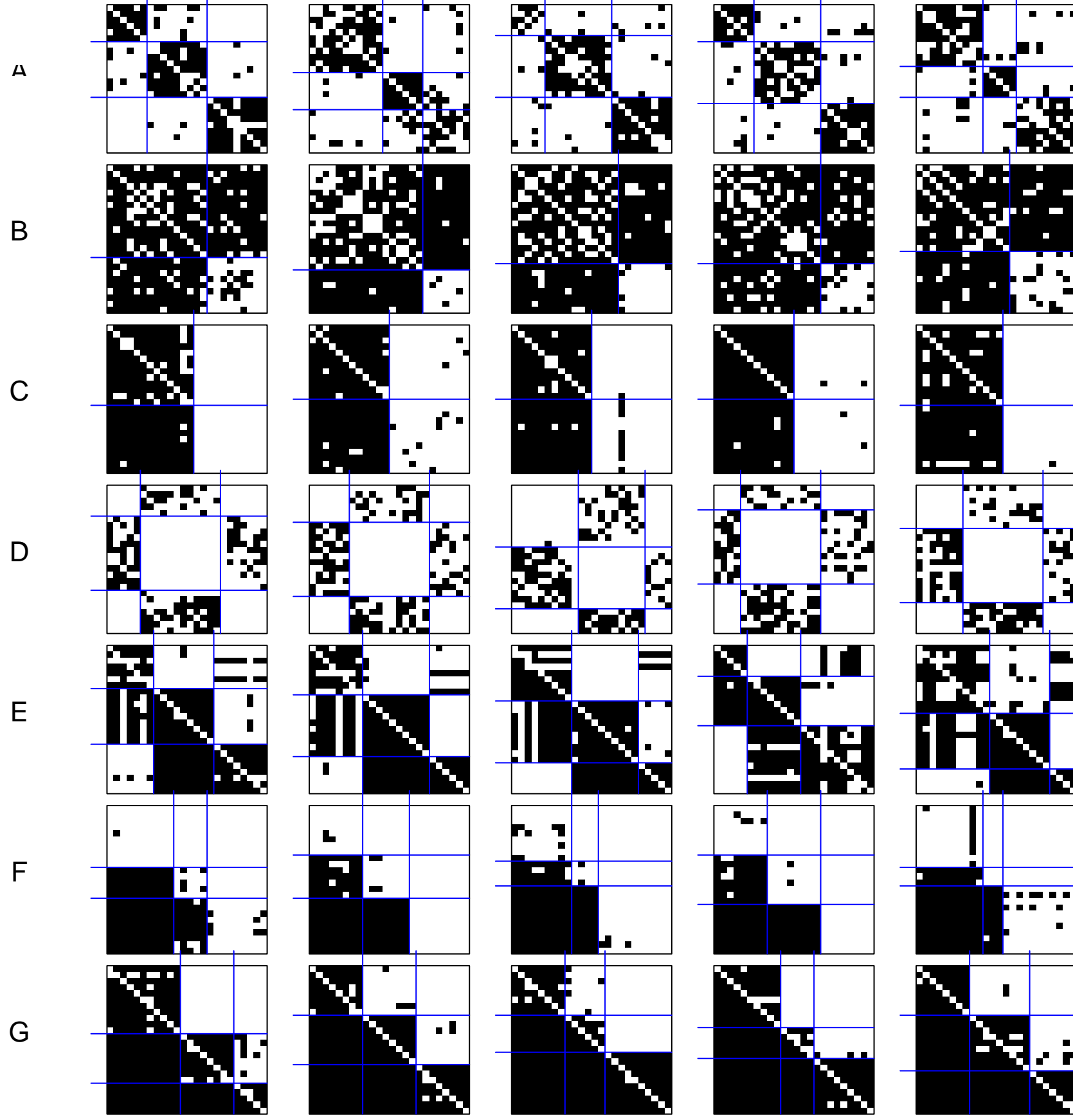

Supplement: S16 Fig — By rows: (A) cohesive; (B) symmetric core-periphery; (C) asymmetric core-periphery; (D) hierarchical without complete blocks on the diagonal; (E) hierarchical with complete blocks on the diagonal; (F) transitivity without complete blocks on the diagonal; (G) transitivity with complete blocks on the diagonal. (PDF) [file pone.0197514.s018.pdf]

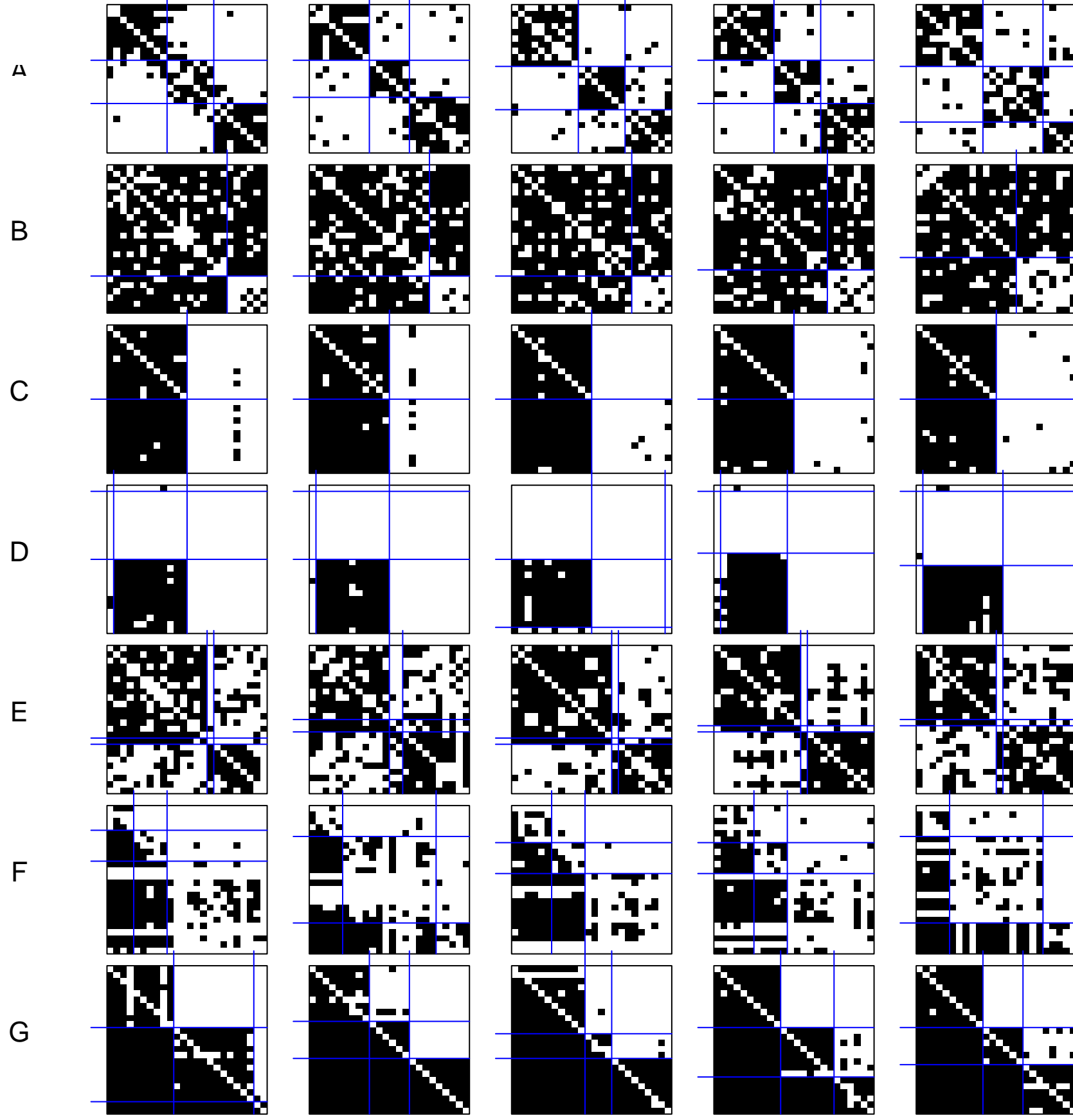

Supplement: S17 Fig — By rows: (A) cohesive; (B) symmetric core-periphery; (C) asymmetric core-periphery; (D) hierarchical without complete blocks on the diagonal; (E) hierarchical with complete blocks on the diagonal; (F) transitivity without complete blocks on the diagonal; (G) transitivity with complete blocks on the diagonal. (PDF) [file pone.0197514.s019.pdf]

H

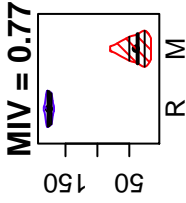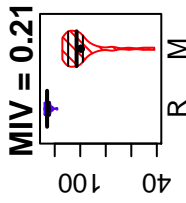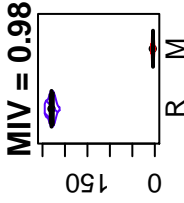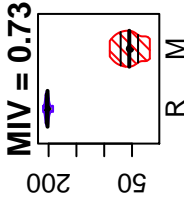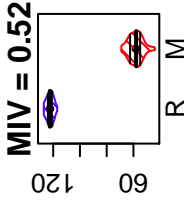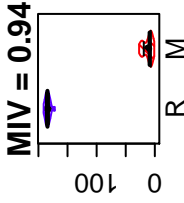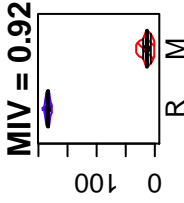

I

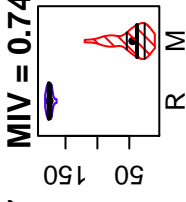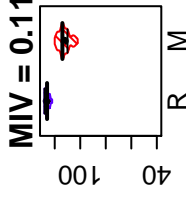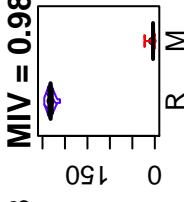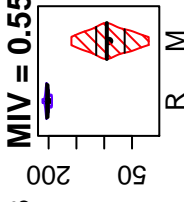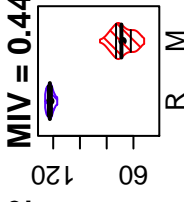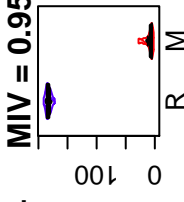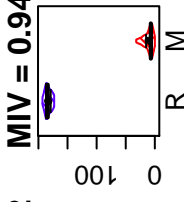

J

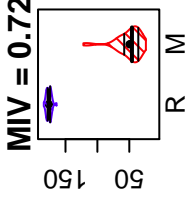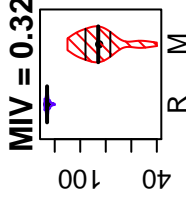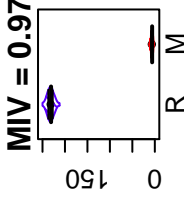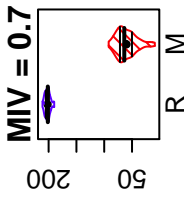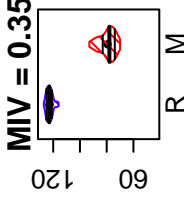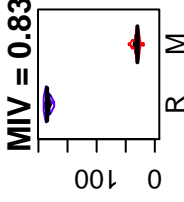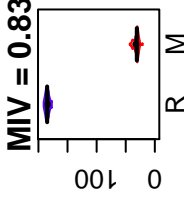

K

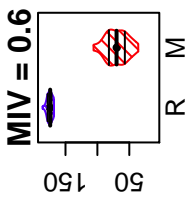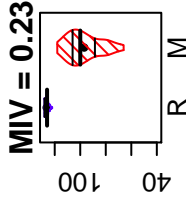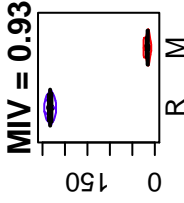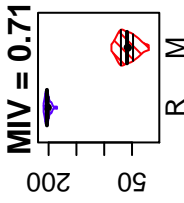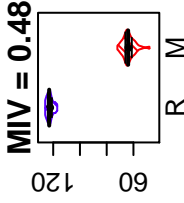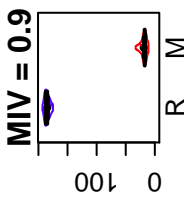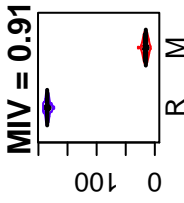

L

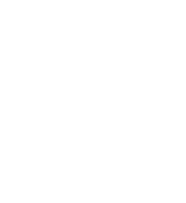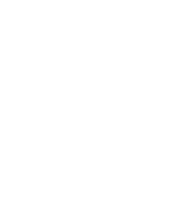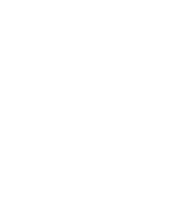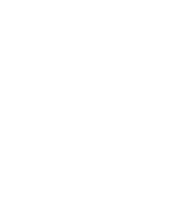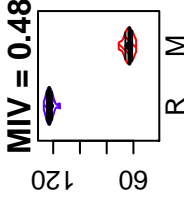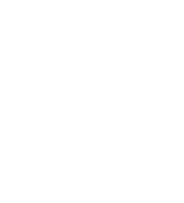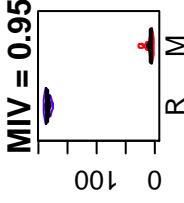

M

Supplement: S19 Fig — R denotes the distribution of PR and M denotes the distribution of Pm. By rows: (A) cohesive; (B) symmetric core-periphery; (C) asymmetric core-periphery; (D) hierarchical without complete blocks on the diagonal; (E) hierarchical with complete blocks on the diagonal; (F) transitivity without complete blocks on the diagonal; (G) transitivity with complete blocks on the diagonal. By columns: (H) all; (I) all allowed; (J) all forbidden; (K) selected; (L) selected allowed; (M) selected forbidden types of triads. (PDF) [file pone.0197514.s021.pdf]

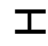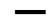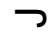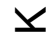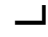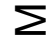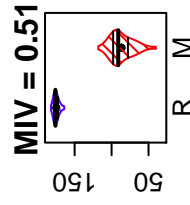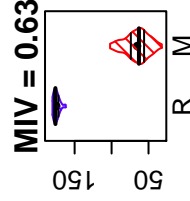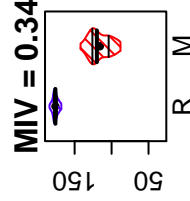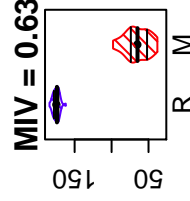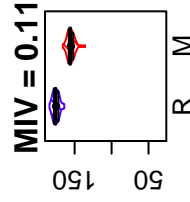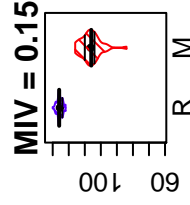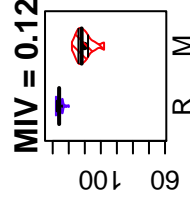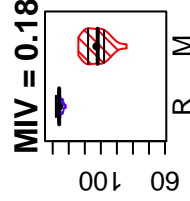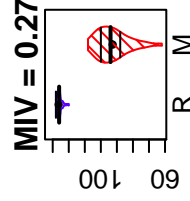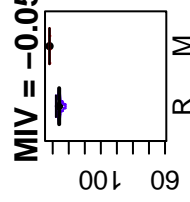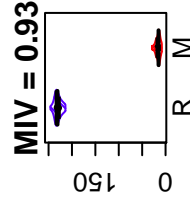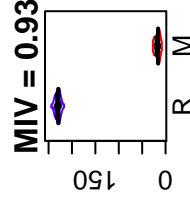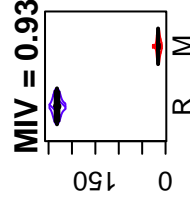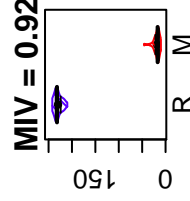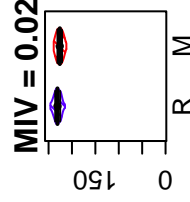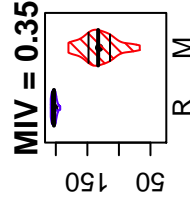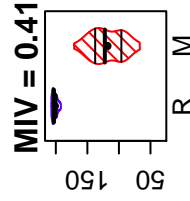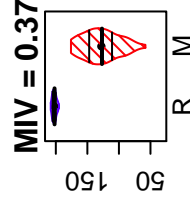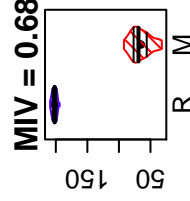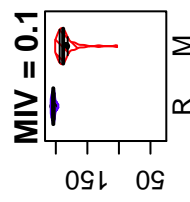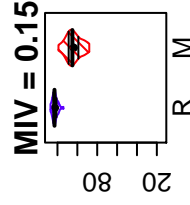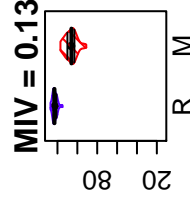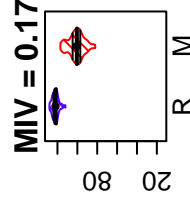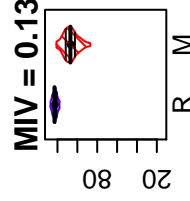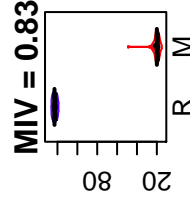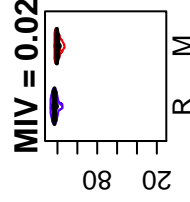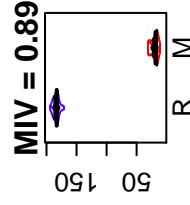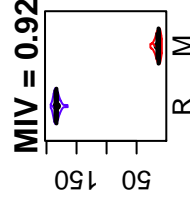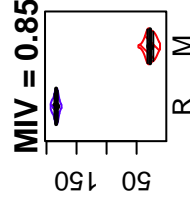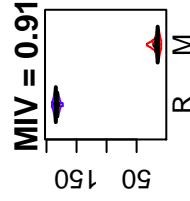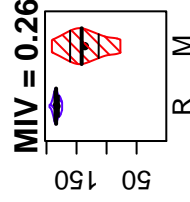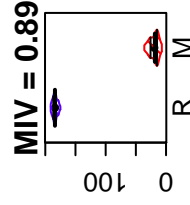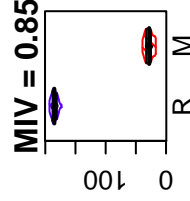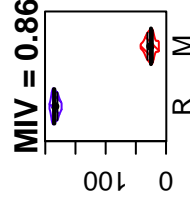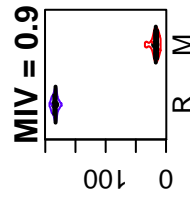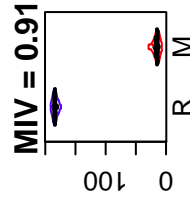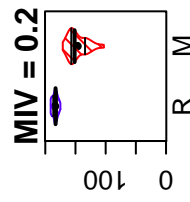

Supplement: S20 Fig — R denotes the distribution of PR and M denotes the distribution of Pm. R denotes the distribution of PR and M denotes the distribution of Pm. By rows: (A) cohesive; (B) symmetric core-periphery; (C) asymmetric core-periphery; (D) hierarchical without complete blocks on the diagonal; (E) hierarchical with complete blocks on the diagonal; (F) transitivity without complete blocks on the diagonal; (G) transitivity with complete blocks on the diagonal. By columns: (H) all; (I) all allowed; (J) all forbidden; (K) selected; (L) selected allowed; (M) selected forbidden types of triads. (PDF) [file pone.0197514.s022.pdf]

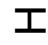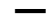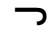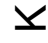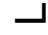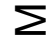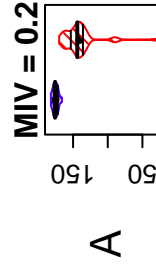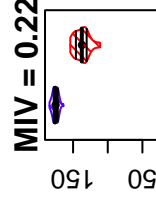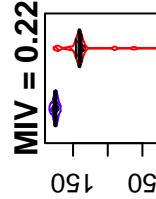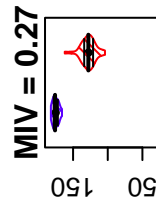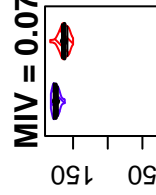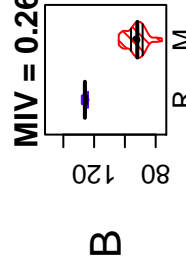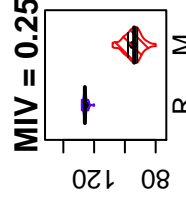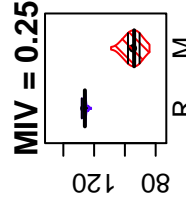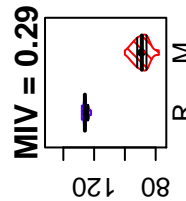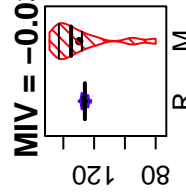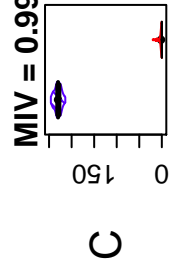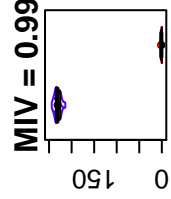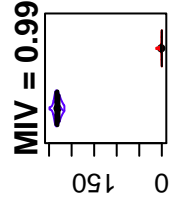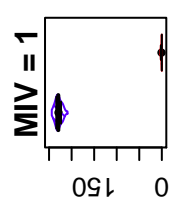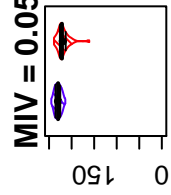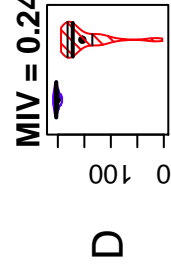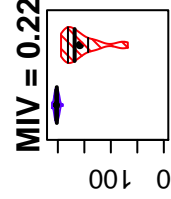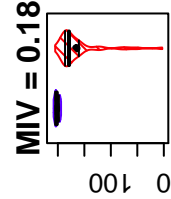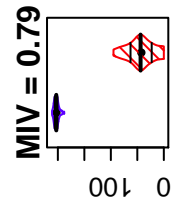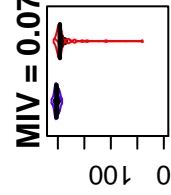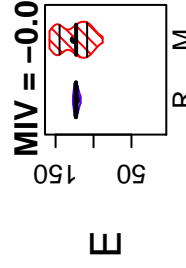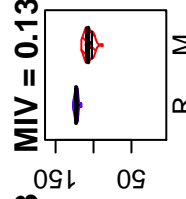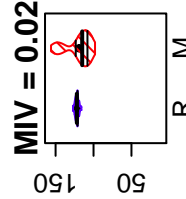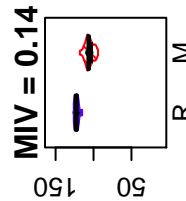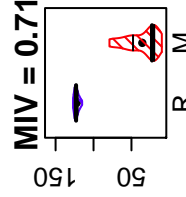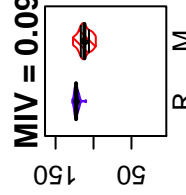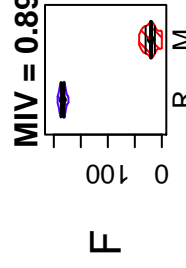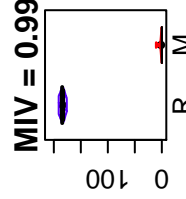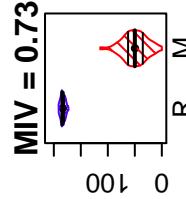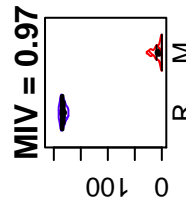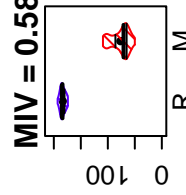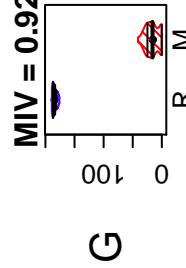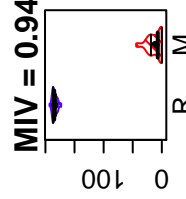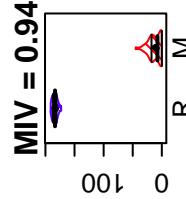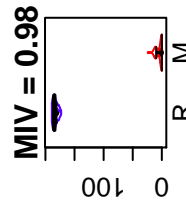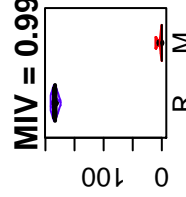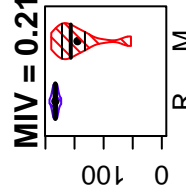

Supplement: S21 Fig — R denotes the distribution of PR and M denotes the distribution of Pm. R denotes the distribution of PR and M denotes the distribution of Pm. By rows: (A) cohesive; (B) symmetric core-periphery; (C) asymmetric core-periphery; (D) hierarchical without complete blocks on the diagonal; (E) hierarchical with complete blocks on the diagonal; (F) transitivity without complete blocks on the diagonal; (G) transitivity with complete blocks on the diagonal. By columns: (H) all; (I) all allowed; (J) all forbidden; (K) selected; (L) selected allowed; (M) selected forbidden types of triads. (PDF) [file pone.0197514.s023.pdf]
