# Supplementary material for: Generating global network structures by triad types
Source: PLoS One. 2018 May 30;13(5):e0197514. doi: 10.1371/journal.pone.0197514 (PMC5976167; doi:10.1371/journal.pone.0197514)
Supplement: S18 Fig — By rows: (A) cohesive; (B) symmetric core-periphery; (C) asymmetric core-periphery; (D) hierarchical without complete blocks on the diagonal; (E) hierarchical with complete blocks on the diagonal; (F) transitivity without complete blocks on the diagonal; (G) transitivity with complete blocks on the diagonal. (PDF) [file pone.0197514.s020.pdf]

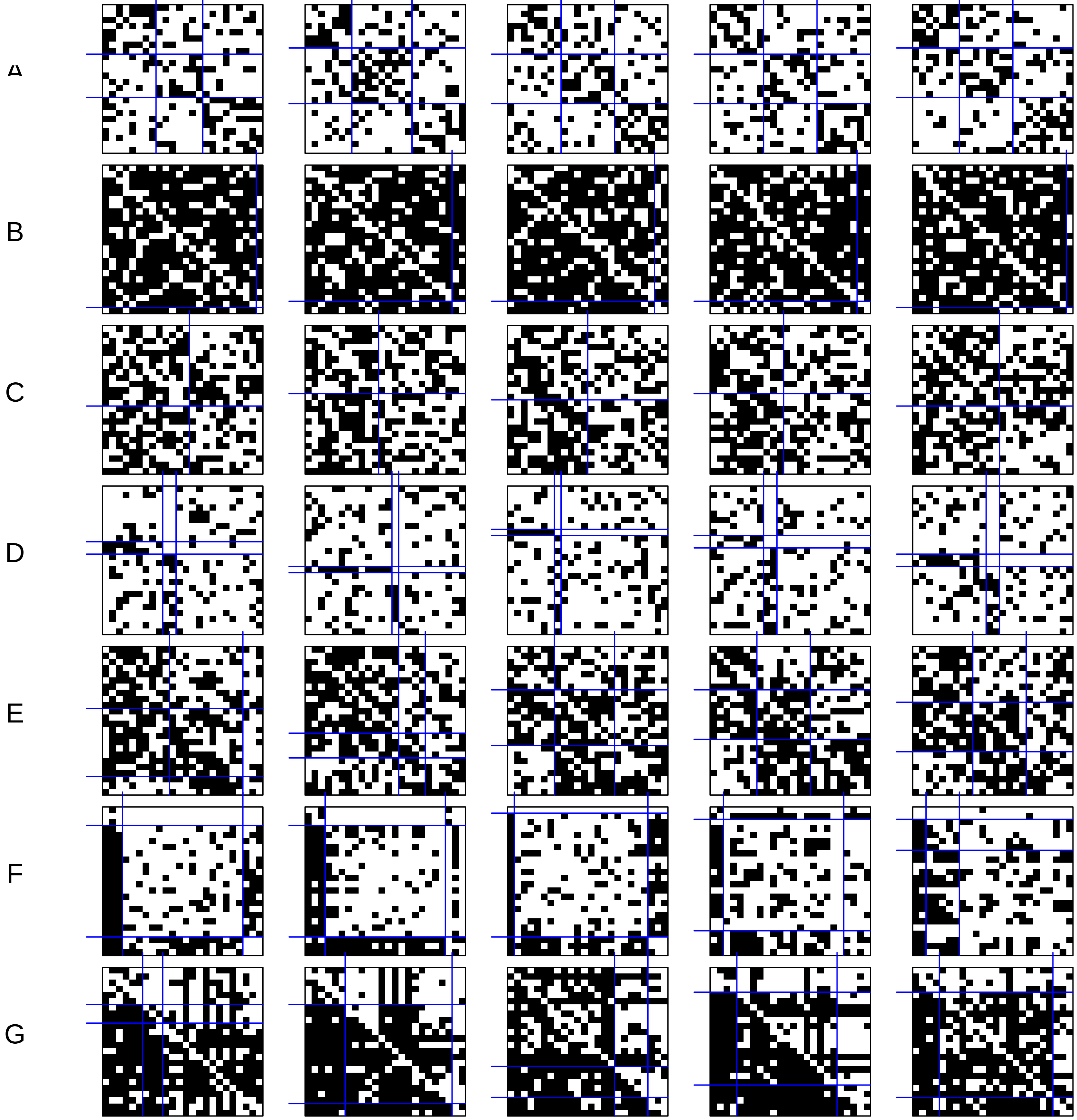
A
B
C
D
E
F
G
